# Complementary Roles of DNA Methylation and miRNA in Regulating Gene Expression Under Environmental Stress in Biological Invasions

**DOI:** 10.1111/eva.70178

**Published:** 2025-11-08

**Authors:** Weijie Yan, Ruiying Fu, Xuena Huang, Aibin Zhan

**Affiliations:** ^1^ Research Center for Eco‐Environmental Sciences Chinese Academy of Sciences Beijing China; ^2^ University of Chinese Academy of Sciences Chinese Academy of Sciences Beijing China

**Keywords:** biological invasion, DNA methylation, environmental challenge, epigenetics, functional complementation, microRNA

## Abstract

Biological invasions significantly threaten global biodiversity and disrupt the stability of ecosystems worldwide. Effective responses to environmental stressors are crucial for invasion success; however, the underlying epigenetic regulatory mechanisms remain poorly understood, especially regarding the interplay among multiple regulatory layers such as DNA methylation and microRNAs (miRNAs). Here we employed an integrative multi‐omics approach to investigate the model invasive *Ciona robusta* subjected to repeated salinity stress. Focusing on canonical osmotic regulation pathways, we revealed a dynamic and coordinated regulation of stress‐responsive gene expression, with miRNAs and DNA methylation playing distinct yet complementary roles across functional pathways/genes and distinct regions within the same genes. Regulating osmolyte shifts during repeated stress, miRNAs emerged as dominant regulators through widespread and flexible targeting of genes, whereas DNA methylation contributed more selectively. Notably, both mechanisms co‐regulated certain genes via spatially distinct genomic regions, supporting a multilayered model of gene regulation. Furthermore, we observed significantly reduced methylation levels in miRNA‐targeted genes, suggesting an evolutionary structural complementarity between the two epigenetic systems. Moreover, the permutation test revealed that dual regulation was a non‐random event. Interestingly, miRNAs and DNA methylation did not converge on a limited set of stress‐related pathways; instead, they provided complementary regulation across multiple functions, while dual regulation did not directly amplify gene expression changes. Together, these findings underscore the critical role of complex interplay among epigenetic processes in enabling rapid phenotypic plasticity and provide novel insights into the molecular mechanisms underlying invasion success under environmental stress.

## 1 Introduction

Biological invasions cause significant global economic losses and ecological damage (Anton et al. 2019; Diagne et al. 2021), ranking among the leading drivers of recent species extinctions, altering ecosystem composition and functioning, and even posing public health risks (VilÀ et al. 2021). Aquatic ecosystems, which provide critical ecosystem services, are especially vulnerable due to anthropogenic pressures and environmental changes (Blakeslee et al. 2020; Turbelin et al. 2024). Despite substantial management efforts, particularly in North America and Oceania, outcomes often remain suboptimal (Cuthbert et al. 2022). This pattern of “intensive effort but limited efficiency” largely stems from an incomplete understanding of the ecological and evolutionary processes involved in biological invasions, particularly the underlying mechanisms that drive invasion success.

During invasions, invasive species often face rapid and recurring environmental fluctuations that threaten survival and constrain habitat expansion (Briski et al. 2013). The ability to respond swiftly to stressors is therefore critical for invasion success, particularly in early establishment and spread (Li et al. 2020). Among adaptive mechanisms, epigenetic processes, such as epigenetic modifications and non‐coding RNA regulation, enhance fitness under stressful conditions (Jonsson and Jonsson 2019), providing faster and more flexible responses than slower evolutionary processes such as natural selection. Notably, microRNA (miRNA) regulation and DNA methylation enable rapid adjustment to environmental challenges (Kooke et al. 2015). For instance, DNA methylation modulates genes involved in water transport, energy, and lipid metabolism (e.g., *aquaporin* and *pnala8*), facilitating ascidian invasions (Fu, Huang, Chen, et al. 2021), while miRNAs regulate stress‐response genes, such as leucine‐rich repeat receptor‐like kinases, supporting the successful invasions of *Solidago canadensis* (Xu et al. 2019).

As key regulators of the transcriptome and fundamental components of gene regulatory networks, miRNAs function independently or interact with other regulatory elements, such as long non‐coding RNAs, to modulate target gene expression post‐transcriptionally (Paraskevopoulou and Hatzigeorgiou 2016; Solís et al. 2022). Typically, miRNAs regulate gene expression by binding to the 3′ Un‐Translated Region (UTR) of target mRNAs, thereby fine‐tuning their expression (Bartel 2009). However, recent studies have revealed a more diverse pattern of miRNA‐mediated gene regulation, showing that miRNAs can bind not only to 5′ UTR and Coding DNA Sequence (CDS) but also modulate gene expression by either repressing or enhancing it (Pu et al. 2019; Xiao et al. 2017). Through these multidimensional interactions, miRNAs establish complex regulatory networks that work closely with other molecular elements and biological processes to enable precise and rapid responses to environmental stresses.

In addition to miRNAs, DNA methylation is a critical epigenetic mechanism that regulates gene expression by modifying the methylation status of cytosine residues (Yang et al. 2014). Such methylation modifications, particularly those in promoters, can repress transcription by hindering the binding of transcription factors or by recruiting methyl‐binding proteins that form repressive chromatin complexes (Greenberg and Bourc'his 2019). Furthermore, DNA methylation can establish or maintain heterochromatin, further silencing gene activity (Rountree and Selker 2010). Recent studies have shown that DNA methylation patterns can be rapidly reprogrammed in response to environmental stresses, with genome‐wide changes occurring rapidly, such as in only 1 h of exposure, in both invasive and nonnative species (Huang et al. 2017; Ozaki et al. 2017). These rapid DNA methylation dynamics play a pivotal role in modulating the expression of stress‐responsive genes. For instance, alterations in methylation levels have been linked to the regulation of genes involved in pollutant detoxification, osmotic stress adaptation, and hypoxic response in fishes (Gao et al. 2025; Huang et al. 2025; Park et al. 2020). Similar regulatory effects have been observed in genes associated with thermal tolerance in oysters (Roberto et al. 2021), microplastic‐induced stress in mussels (Park et al. 2024), and acid–base homeostasis in sea urchins under pH stress (Strader et al. 2019). These findings underscore the role of DNA methylation as an efficient regulatory mechanism for fine‐tuning gene expression, enabling organisms to respond rapidly to environmental challenges.

Despite significant progress in understanding miRNA and DNA methylation individually, their interplay in regulating gene expression under environmental challenges remains largely unexplored. Emerging evidence suggests potential functional interactions between these two epigenetic mechanisms, although the nature of their relationship appears species‐specific. In humans and mice, for example, genes with stronger DNA methylation tend to have fewer miRNA binding sites, whereas genes targeted by miRNAs generally show lower DNA methylation levels, indicating a complementary relationship (Su et al. 2011b). Conversely, in cattle skeletal muscle, miRNA binding has been reported to promote DNA methylation (Huang et al. 2014). While studies on miRNA‐DNA methylation interactions remain limited, particularly in invasive species, existing findings highlight their potential collaborative role in modulating gene expression in response to environmental stress. A deeper understanding of these dynamics could shed light on the adaptability and resilience of invasive species and inform the development of more effective management strategies.

Given the complex regulatory networks of miRNAs and DNA methylation, choosing an appropriate model species is crucial for studying gene expression responses to environmental stress during invasions. *Ciona robusta* (formerly sp. A of the *C. intestinalis* complex) is presumably native to the Northwest Pacific but has spread globally (Brunetti et al. 2015; Chen et al. 2018). Its small, well‐sequenced genome (~120 Mb), high invasiveness, environmental tolerance, and adaptive capacity make it a valuable model in invasion science (Satou et al. 2019; Therriault and Herborg 2008; Zhan et al. 2015). Previous studies revealed that *C. robusta* triggered gene expression, DNA methylation modification, and miRNA regulation to react environmental stress (Huang et al. 2017; Fu, Huang, Chen, et al. 2021; Yan et al. 2025). More interestingly, miRNA‐mediated regulation was linked to shifts in free amino acid metabolism & biogenesis and ion transport to enable osmotic balance during stress challenge (Yan et al. 2025). These rapid and multi‐layered functional regulatory responses highlight *Ciona* ascidians as an ideal model for investigating the interplay between miRNAs and DNA methylation in the regulation of gene expression under environmental stress.

Using the model invasive ascidian *C. robusta* in this study, we simulated the repeated hypersalinity stress encountered during the invasion process and integrated transcriptomic, miRNAomic, and DNA methylation data within a laboratory‐controlled experimental system. Salinity is a critical environmental factor influencing survival (Tuwo et al. 2020), development (Melo et al. 2019), growth (Li and Li 2010), and reproduction (Neves et al. 2019) of marine organisms, making it one of the most important factors determining the success of biological invasions in coastal and marine ecosystems (Grabowski et al. 2007). Given the crucial role of salinity in shaping invasion outcomes and the complexity of physiological responses required to cope with such stress, a comprehensive investigation into the underlying molecular mechanisms is essential. In this context, our objectives in this study are to: (1) investigate the transcriptomic response patterns to repeated salinity stress through canonical strategies, and (2) explore the functional interactions between two key epigenetic mechanisms (miRNAs and DNA methylation) in regulating gene expression responses. Based on previous insights into the interaction between DNA methylation and miRNA regulation, we aim to test the hypothesis that miRNAs and DNA methylation can mutually regulate each other's functional genes and likely play complementary roles in controlling gene expression. The results are expected to provide valuable insights into the strategies employed by invaders under repeated environmental stress and the functional interplay between miRNA regulation and DNA methylation in facilitating invasion success.

## 2 Materials and Methods

### 2.1 Experimental Design, Sample Collection, Sequencing and Multi‐Omics Analysis

Adult *C. robusta* specimens were collected from an aquaculture facility in Dalian, Liaoning Province, China (38°49′19″ N, 121°2′28″ E). Throughout the experiment, individuals were fed with *Spirulina* sp. and *Chlorella* sp., with only healthy individuals selected for subsequent stress. To simulate the salinity stress encountered during the invasion process, *C. robusta* was exposed to recurrent hyperosmotic stress, following protocols established in our previous studies on gene expression, DNA methylation, and miRNA response patterns under environmental challenges (Fu, Huang, Chen, et al. 2021; Fu, Huang, and Zhan 2021; Li et al. 2020). In general, the control and recovery salinity levels were maintained at 30‰, corresponding to the natural conditions at the sampling site, while the hyperosmotic condition was set at 40‰ by adding sea salt to seawater, reflecting the high salinity within the invasive ranges of *C. robusta* such as the Red Sea (Chen et al. 2018). After acclimation in the laboratory, six healthy individuals were designated as the control group. The remaining individuals were exposed to hyperosmotic conditions (40‰) for 72 h (Stress stage 1, S1), with samples collected at 24 and 48 h. Following this, individuals were transferred back to 30‰ salinity for 24 h (Recovery stage, R), and samples were collected at 96 h. Finally, individuals were re‐exposed to 40‰ salinity for an additional 72 h (Stress stage 2, S2), with samples collected at 120 and 144 h (Figure S1). The sampling time points were strategically designed and selected to correspond with key stages of transcriptomic responses to salinity stress, and the biological replicate numbers of 24, 48, 96, 120, and 144 h were four, six, four, four, and three, respectively after quality control of RNAs (Fu, Huang, Chen, et al. 2021; Fu, Huang, and Zhan 2021; Huang et al. 2018, 2017; Li et al. 2020).

The tissue collection and high‐throughput sequencing procedures were detailed in our previous studies (Fu, Huang, Chen, et al. 2021; Yan et al. 2025). The raw mRNA and DNA methylation sequencing data have been deposited in the NCBI Sequence Read Archive (SRA) under accession number PRJNA775866, and the raw miRNA‐seq data are available under accession number PRJNA1120970 (Fu, Huang, Chen, et al. 2021; Yan et al. 2025).

To reveal the expression levels of miRNAs, the analysis of miRNA‐seq data was performed as described in our previous study (Yan et al. 2025). In brief, miRNA expression levels at each sampling point were compared to those at 0 h. Differentially expressed miRNAs (DEmiRNAs) were identified using a threshold of |log_2_(fold change)| > 1 and adjusted *p*‐value < 0.05. Target genes of the DEmiRNAs were predicted using both RNAhybrid and miRanda algorithms, applying an energy threshold of ≤ −20 kcal/mol. Only overlapping targets from both prediction tools were considered as true targets (Betel et al. 2008; Rehmsmeier et al. 2004). The expression correlation between miRNAs and their corresponding target genes was assessed using the Pearson correlation coefficient, calculated with the ‘psych’ package in R (Revelle and Condon 2019), and the distribution of miRNA binding sites across the 3′ untranslated region (3′UTR), 5′ untranslated region (5′UTR), and coding sequence (CDS) was analyzed using the ‘GenomicRanges’ package in R. (Lawrence et al. 2013). For transcriptomic analysis, quality control was performed using FastQC v0.11.9 (https://www.bioinformatics.babraham.ac.uk/projects/fastqc/). The raw reads were mapped to the reference genome of *C. robusta* using Hisat2 v2.1.0, read counts were obtained using featureCounts, and differential gene expression analysis was conducted with the DESeq2 package, applying the same significance criteria of adjusted *p*‐value < 0.05 and |log_2_(fold change)| > 1 (Liao et al. 2013; Love et al. 2014; Kim et al. 2019). Genes identified as differentially expressed were referred to as differentially expressed target genes (DETGs).

For DNA methylation data analysis, the raw data was trimmed and mapped to the genome using Trimmomatic (Bolger et al. 2014) and FastQC (https://www.bioinformatics.babraham.ac.uk/projects/fastqc/), respectively. Subsequently, we used Bismark 2.0 to extract the methylation status of individual cytosines (Krueger and Andrews 2011). Methylation levels at individual CpG sites were processed using the methylKit package in R (Akalin et al. 2012). Differentially methylated sites were identified by comparing the control group (0 h) with other sampling points, as well as the recovery stage (96 h) with the second hyperosmotic stress stage (S2; 120 and 144 h). Genes containing differentially methylated sites within their gene body or promoter regions were defined as DNA methylation‐related stress‐responsive genes (DMSGs). The gene body was defined as the region from the transcription start site (TSS) to the transcription end site, and the promoter region as the 2000 bp upstream of the TSS (Fu, Huang, Chen, et al. 2021; Fu, Huang, and Zhan 2021).

### 2.2 Identification of Canonical Osmotic Stress‐Related Genes, and Expression Analysis

Given the complexity of salinity stress responses in marine species including *Ciona* (Fu, Huang, Chen, et al. 2021; Huang et al. 2023; Pagano et al. 2022), as well as the largely unknown upstream and downstream regulatory networks of osmotic stress‐related genes, this study specifically focused on all canonical osmotic stress‐related genes. Genes involved in the three canonical strategies of cellular osmotic homeostasis in aquatic animals, including free amino acid (FAA) metabolism and biosynthesis, water transport, and ion transport, were retrieved from the *C. robusta* genome. These genes were annotated based on previous studies in all available aquatic animals (Cao and Shi 2019; Liu et al. 2019; Pu and Zhan 2017; Verri et al. 2012). Differentially expressed genes (DEGs) associated with these pathways were identified from the transcriptomic data and selected for further analysis. The expression levels of all the functional genes were visualized by ‘pheatmap’ package in R (Kolde and Kolde 2015).

### 2.3 Interplay Between DNA Methylation and miRNAs in Regulating Stress‐Responsive Genes

To investigate the interplay between DNA methylation and miRNAs in regulating gene expression, the overlap of gene regulation by these two mechanisms was visualized using a pie chart generated with the online OmicShare tools (Mu et al. 2024). Functional genes regulated solely by DNA methylation, solely by miRNAs, or jointly by both were listed along with their expression patterns. Correlations between miRNA expression and target gene expression, as well as between DNA methylation level and gene expression, were assessed using the Pearson correlation coefficient with the psych package in R (Revelle and Condon 2019) and visualized using distinct colors and shapes.

To further evaluate the potential causes of the coordinated regulation (see the Section 3 for more detail) (i.e., whether miRNA‐targeted genes lack methylation sites), we compared the global DNA methylation levels of miRNA‐targeted and non‐miRNA‐targeted genes; we compared global DNA methylation levels between miRNA target genes and genes not directly regulated by differentially expressed miRNAs (DEmiRNAs). This comparison was conducted using the Wilcoxon test and visualized as a box plot with the ‘ggplot2’ package in R (Wilkinson 2011). Additionally, to assess potential structural divergence, we compared the number of DNA methylation sites between miRNA target genes and functional genes not directly regulated by miRNAs, also using the Wilcoxon test and visualized accordingly.

In addition, to further validate the functional interplay between DNA methylation and miRNA regulation, permutation tests were performed to assess whether dual regulation occurred randomly. These tests involved 1000 iterations using the same number of miRNA‐regulated and DNA methylation‐regulated genes. Furthermore, GO enrichment analysis of dually regulated genes was conducted using the online OmicShare tools (Mu et al. 2024). The |log_2_(fold change)| levels of three gene groups, including (i) miRNA‐only regulated, (ii) DNA methylation‐only regulated, and (iii) dually regulated, were visualized using boxplots in R. Differences between group (i) versus group (iii) and group (ii) versus group (iii) were assessed using the Wilcoxon test, with a significance threshold set at 0.05.

## 3 Results

### 3.1 Dynamic Responsive Expression of miRNAs and Target Genes

**FIGURE 1:**
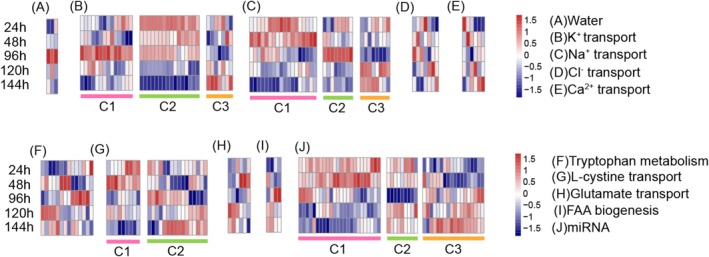
Heatmap of differentially expressed functional genes involved in water transport, ion transport, and free amino acid (FAA) metabolism and biogenesis, along with miRNAs regulating these genes. (A) Expression of genes related to water transport; (B–E) expression of genes related to ion transport; (F–I) expression of genes associated with FAA metabolism and biogenesis; (J) expression of relevant miRNAs.

To illustrate the responsive pattern of canonical strategy genes, a total of three, 49, and 96 DEGs related to water transport, FAA metabolism and biogenesis, and ion transport, respectively, were identified in the *C. robusta* genome (Table S1). Following high‐salinity stress, we observed an upregulation of genes associated with water transport (Figure 1A). Potassium (K^+^) transport genes were divided into three expression patterns: Cluster 1 (C1) genes showed increased expression at 96 h; Cluster 2 (C2) genes were upregulated at the initial stress stage but declined to relatively low levels thereafter; and Cluster 3 (C3) genes were downregulated initially and then upregulated (Figure 1B). Sodium (Na^+^) transport genes also formed three clusters: C1 genes exhibited an initial increase followed by a decrease; C2 genes showed elevated expression at 96 h; and C3 genes were initially downregulated and subsequently upregulated (Figure 1C). Interestingly, chloride (Cl^−^) and calcium (Ca^2+^) transport genes displayed distinct response profiles, reflecting diverse regulatory patterns even among genes with similar functions (Figure 1D,E). Genes involved in FAA metabolism and biogenesis exhibited diverse expression patterns. L‐cystine transport genes were grouped into two clusters: Cluster 1 (C1) genes showed an initial increase followed by a decrease in expression, while Cluster 2 (C2) genes exhibited more variable response patterns (Figure 1G). Additionally, other FAA‐related genes, including those involved in tryptophan metabolism, glutamate transport, and general FAA metabolism, displayed distinct, gene‐specific response profiles (Figure 1F,H,I). Such pathway‐ and gene‐specific responses to high‐salinity stress underscore the dynamic and diverse regulatory mechanisms involved in stress adaptation, reflecting coordinated yet distinct transcriptional programs that contribute to osmotic homeostasis.

In total, 51 DEmiRNAs were involved in the regulation of ion transport and FAA metabolism and biogenesis genes, while water transport‐related genes were not directly regulated by DEmiRNAs. Interestingly, the expression levels of DEmiRNAs were divided into three distinct patterns: Cluster 1 (C1) exhibited upregulation followed by downregulation over the stress duration, Cluster 2 (C2) showed downregulation during the recovery stage (R) and upregulation at the stress stages (S1 and S2), and Cluster 3 (C3) demonstrated a decrease in expression at the initial stage followed by upregulation (Figure 1J). The distinct expression pattern indicates that miRNA expression responds in a synchronized manner during high‐salinity stress, contributing to the regulation of gene expression networks.

### 3.2 Exclusive Regulation of miRNA and DNA Methylation on Gene Expression

**FIGURE 2:**
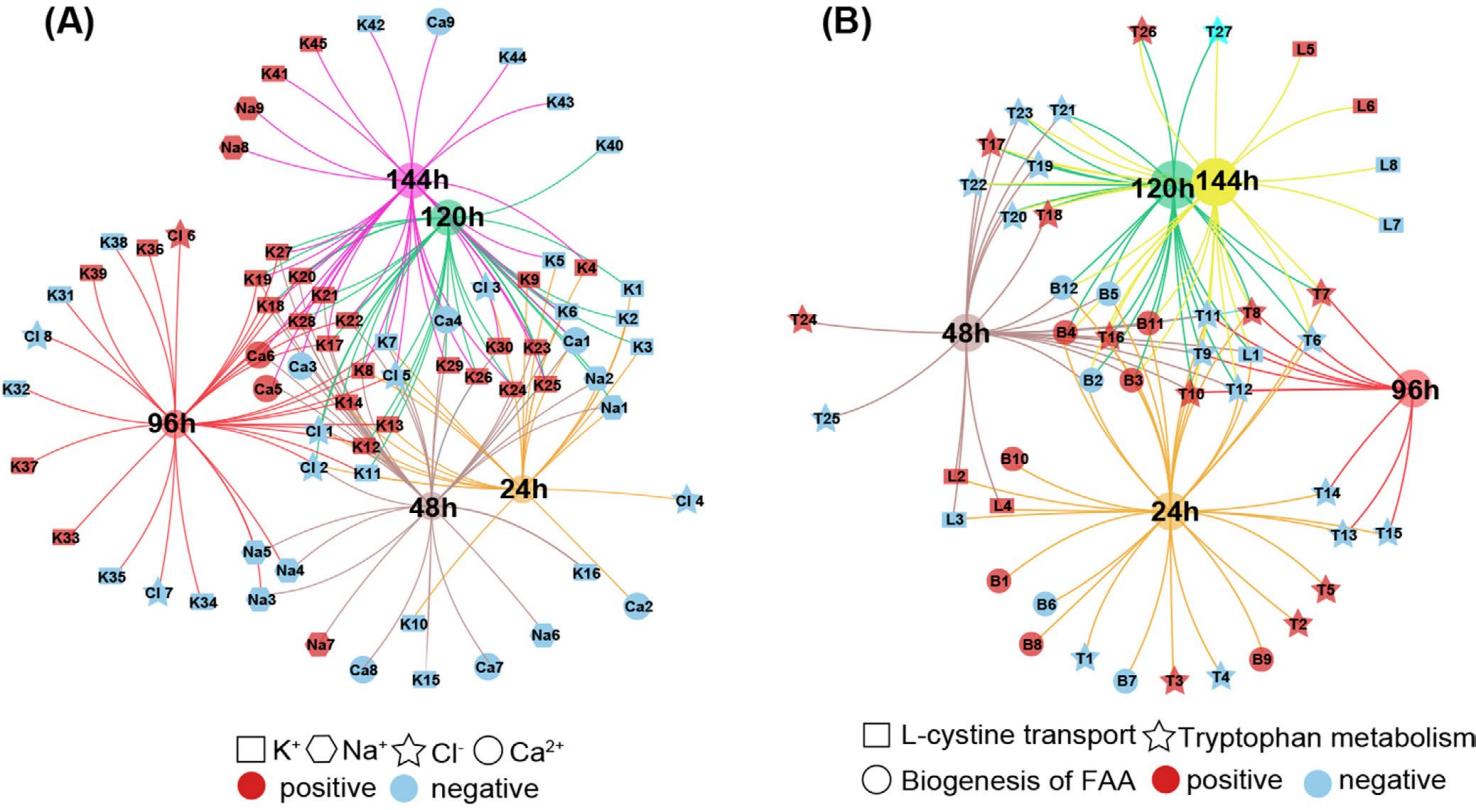
Specific and shared miRNA‐target pairs at different sampling time points (24, 48, 96, 120, and 144 h). (A) Target pairs related to ion transport, with K^+^, Na^+^, Cl^−^, and Ca^2+^ labeled as square, hexagon, five‐pointed star, and circle, respectively. Positive and negative correlations are indicated by red and blue, respectively. (B) Target pairs related to free amino acid (FAA) metabolism and biogenesis, with L‐cystine transport, tryptophan metabolism, and FAA biogenesis labeled as square, five‐pointed star, and circle, respectively. Red and blue represent positive and negative correlations, respectively.

The miRNA regulation on target genes exhibited a time‐dependent pattern. In summary, 71 miRNA‐mRNA target pairs related to ion transport and 41 pairs associated with FAA metabolism/biogenesis were identified. Specifically, only three ion transport target pairs were activated at all sampling points (Figure 2A), while six target pairs related to FAA metabolism and biogenesis were triggered at all time points (Figure 2B). Interestingly, different targets were regulated at various sampling points to achieve the same biological functions. For instance, several genes associated with K^+^ transport were regulated at distinct time points: *kcnk10* at 24 h, *kcne5/kcnv1* at 48 h, *kcnq3/kcnk16/kcnip1/hcn1* at 96 h, *kcnh3* at 120 h, and *kcns1/kcns2* at 144 h (Figure 2A and Table S2). A similar pattern was observed in FAA metabolism‐related functional genes, such as *cyp2j2_1* at 24 h and *cyp2u1* at 48 h, both of which are also involved in tryptophan metabolism (Figure 2B and Table S2). The observation of “all roads lead to Rome” reflects compensatory regulation within the biological system, showing that organisms employ diverse strategies to balance and optimize fitness and homeostasis.

To further investigate the regulatory patterns of miRNAs, we analyzed the binding sites of miRNAs and the correlation between miRNAs and their corresponding targets. For ion transport‐related functional genes, the majority of binding sites were located in CDS, accounting for approximately 67% of the total sites. The 3′UTR and 5′UTR regions represented 23% and 10% of the binding sites, respectively (Figure S2A). Notably, a higher negative correlation was observed in the UTR regions, with 73% of the binding sites in the 3′UTR and 70% in the 5'UTR exhibiting negative correlations. In contrast, the CDS region showed a lower negative correlation (42%) (Figure S2A). A similar distribution of binding sites was observed for free amino acid (FAA)‐related functional genes, with 74% of the binding sites in the CDS, and 23% and 3% in the 3′UTR and 5′UTR, respectively. The correlation between miRNAs and their targets in the CDS and 3'UTR regions showed similar proportions of positive and negative correlations, while the 5′UTR binding sites predominantly exhibited negative correlations (Figure S2B). This pattern indicates that miRNA‐mediated negative regulation is primarily achieved through binding to the UTR regions, while targeting the CDS involves both negative and positive regulation.

For DNA methylation patterns, we found a trend similar to that observed in miRNA regulation: water transport genes were not affected by DNA methylation. In contrast, 14 ion transport genes and eight FAA metabolism/biogenesis genes exhibited differential DNA methylation levels and were classified as DNA methylation‐related stress‐responsive genes (DMSGs), and the proportions of genes with differential DNA methylation were 38% for ion transport and 50% for FAA metabolism/biogenesis, respectively (Table 1). Among DMSGs in ion transport, the majority of DNA methylation occurred within the gene body (64%), with comparable proportions of positive and negative correlations. Similar proportions of positive and negative correlations were also detected in the promoter region. For FAA metabolism/biogenesis genes, the number of methylation sites was equal between gene body and promoter regions, and the correlation between methylation and gene expression, whether positive or negative, was also evenly distributed in both regions (Table 1). This observation suggests that DNA methylation in the gene body plays a regulatory role similar to that in the promoter region.

**TABLE 1:** Differential methylation levels of DNA methylation‐related stress‐responsive genes (DMSGs).

Gene ID	Gene name	Methy site	Location	Diff.methy	Correlation	Function
KY. UAContig47.1	SCN9A_3	UAContig47:280	Promoter	Up	Negative	Sodium transport
KY. Chr9.637	SLC10A2	Chr9:4782170	Gene body	Down	Positive	Sodium transport
KY. Chr9.624	SLC24A2	Chr9:4627670	Gene body	Down	Negative	Calcium/potassium/sodium transport
KY. Chr8.402	SLC20A1	Chr8:2357750	Promoter	Down	Positive	Sodium transport
KY. Chr8.1384	PRDX2	Chr8:8238281	Promoter	Down	Positive	Phenylalanine metabolism
KY. Chr8.177	CYP2J2.1	Chr8:837756	Promoter	Up	Positive	Tryptophan metabolism
KY. Chr7.932	SLC8A1	Chr7:7010203	Gene body	Up	Positive	Calcium/sodium transport
KY. Chr5.634	SCN11A	Chr:4217708	Gene body	Down	Positive	Sodium transport
KY. Chr5.126	AASS	Chr5:983111	Gene body	Up	Negative	Lysine degradation
KY. Chr5.968	KCNK16	Chr5:6604525	Promoter	Up	Positive	Potassium transport
KY. Chr4.1205	SLC12A9	Chr4:6708608	Gene body	Down	Negative	Chlorine/potassium transport
KY. Chr4.1014	KCNIP1	Chr4:5421697	Promoter	Down	Negative	Potassium transport
KY. Chr3.71	SLC12A4	Chr3:398351	Gene body	Down	Positive	Chlorine/potassium transport
KY. Chr3.975	GGT1	Chr3:6523054	Gene body	Up	Positive	Selenoamino acid metabolism
KY. Chr3.311	ALDH3A2	Chr3:1858597	Promoter	Down	Negative	Tyrosine metabolism
KY. Chr2.2019	TRPA1	Chr2:7361442	Promoter	Up	Positive	Calcium transport
KY. Chr2.905	GSTZ1	Chr2:2880336	Promoter	Up	Negative	Glutathione metabolism
KY. Chr11.280	SLC7A8	Chr11:1639491	Gene body	Down	Negative	L‐cystine transport
KY. Chr11.479	KCNJ5	Chr11:3038987	Gene body	Down	Negative	Potassium transport
KY. Chr11.297	CLCN5	Chr11:1739243	Gene body	Up	Negative	Chlorine transport
KY. Chr11.1277	PGAM2.1	Chr11:8300541	Gene body	Down	Positive	Biosynthesis of amino acids
KY. Chr1.507	KCNAB2	Chr1:1525161	Gene body	Down	Positive	Potassium transport

### 3.3 Functional Interplay Between miRNA Regulation and DNA Methylation

**FIGURE 3:**
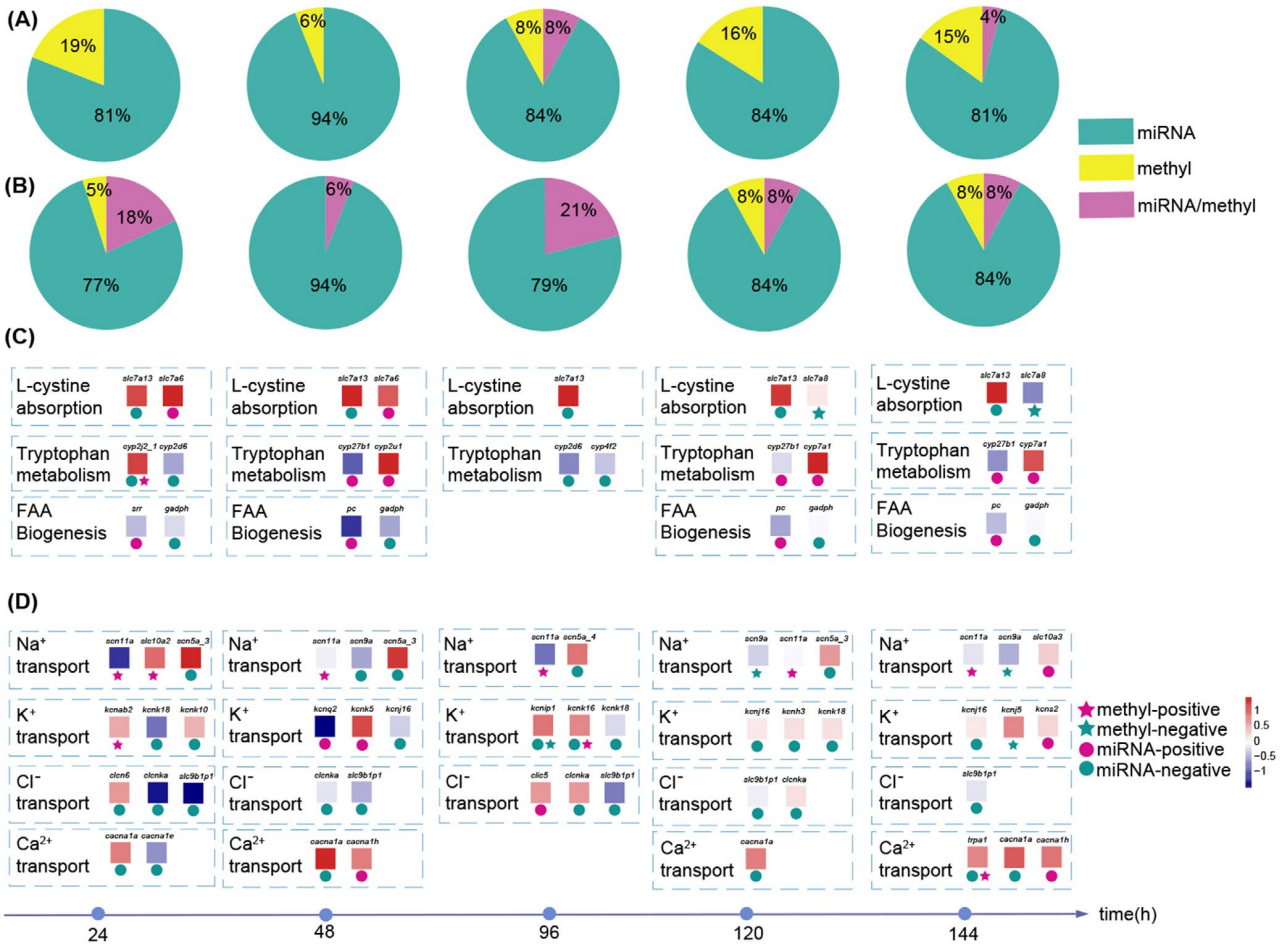
Analysis of the functional relationship between miRNA regulation and DNA methylation. (A) and (B) show the percentage of functional genes involved in ion transport and free amino acid (FAA) metabolism, respectively, regulated by these two mechanisms. Genes regulated solely by miRNAs, DNA methylation, and co‐regulation by both mechanisms are labeled in green, yellow, and purple, respectively. (C) and (D) illustrate the functional genes and their regulation by the two mechanisms during ion transport and FAA metabolism and biogenesis, respectively. Squares represent target gene expression levels, circles represent miRNA regulation, and five‐pointed stars represent DNA methylation regulation. The color of the circles and five‐pointed stars indicates the correlation relationship: Red for positive and green for negative correlation.

To investigate the functional interaction between miRNA regulation and DNA methylation, we examined the genes affected by both mechanisms. Overall, miRNAs regulated a much larger number of genes than DNA methylation in both ion transport and FAA metabolism and biogenesis pathways. For ion transport, more than 80% of genes were solely regulated by miRNAs (Figure 3A). A similar pattern was observed in FAA metabolism and biogenesis genes, where miRNAs exclusively regulated 77% (24 h), 94% (48 h), 79% (96 h), 84% (120 h), and 84% (144 h) of genes (Figure 3B). In contrast, DNA methylation alone regulated only a small proportion of ion transport genes across all sampling points, with 19%, 6%, 8%, 16%, and 15% of genes regulated at 24, 48, 96, 120, and 144 h, respectively (Figure 3A). Similarly, DNA methylation exhibited minimal independent regulation of FAA metabolism and biogenesis genes, with only 5%, 8%, and 8% of genes regulated at 24, 120, and 144 h, respectively (Figure 3B). Co‐regulation by miRNAs and DNA methylation was observed for ion transport genes, with only 8% at 96 h and 4% at 144 h (Figure 3A). A similar pattern was seen for FAA metabolism and biogenesis genes, where co‐regulation occurred at all time points, with 18%, 6%, 21%, 8%, and 8% of genes co‐regulated at 24, 48, 96, 120, and 144 h, respectively (Figure 3B). This finding clearly demonstrates that, despite the co‐regulation of both mechanisms, miRNAs exert a more dominant role in regulating a larger number of genes compared to DNA methylation.

Interestingly, when both miRNA and DNA methylation regulated the same target genes, they acted at distinct gene regions. For example, the ion transport gene *KCNK16* was regulated by both miRNA and DNA methylation. DNA methylation occurred at the *KCNK16* promoter, while miRNA (miR‐4000b‐5p) bound to its CDS, and miR‐7707a‐3p targeted its 3′ UTR (Figure S3A). Similarly, for the FAA‐related gene *cyp2j2_1*, DNA methylation was observed at the promoter, while miR‐124‐2‐5p/miR‐196‐5p bound to its CDS and miR‐2212b‐5p/miR‐4070‐5p/miR‐2f‐5p targeted its 3′ UTR (Figure S3B). In addition to the coordinated regulation of different gene sets by miRNAs and DNA methylation, this finding adds another layer of “coordinated regulation” to their interplay: regulating the same genes at different regions, likely contributing to the fine‐tuning of stress‐responsive genes.

**FIGURE 4:**
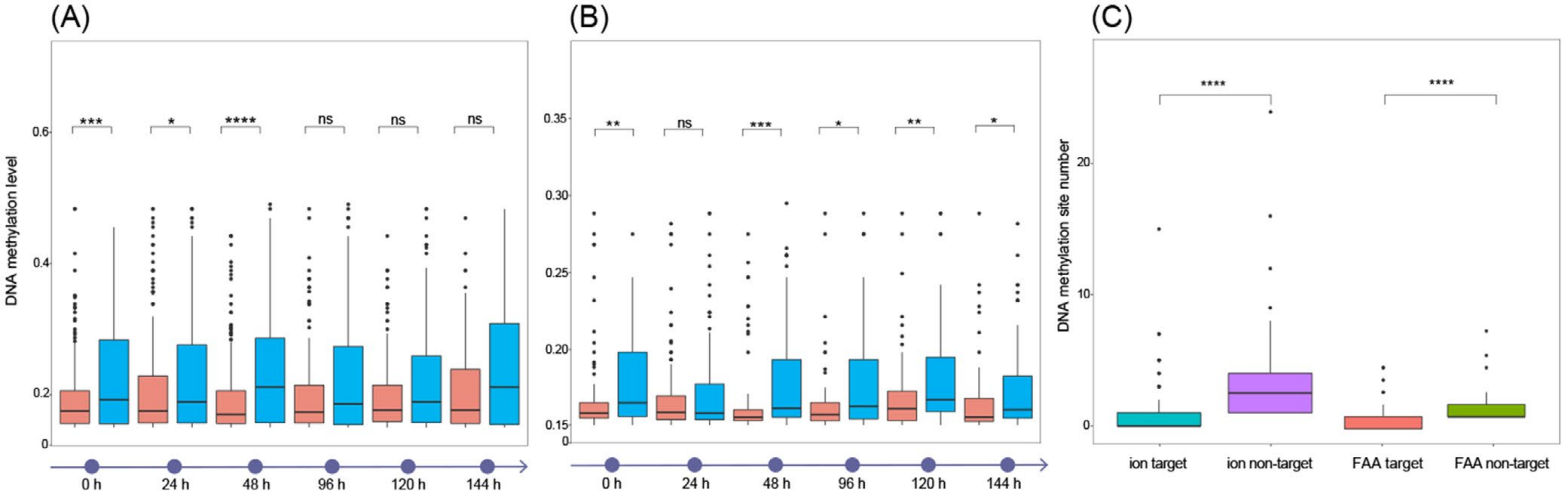
Comparison of global DNA methylation levels between miRNA targets and non‐targets at different sampling time points. (A) Ion transport and (B) free amino acid (FAA) metabolism and biogenesis. Red represents functional genes regulated by miRNAs, while blue represents genes not regulated by miRNAs. (C) Comparison of DNA methylation site numbers, showing the number of DNA methylation sites in miRNA target genes versus non‐target genes. The differences in DNA methylation levels and site numbers between miRNA targets and non‐miRNA targets were assessed using the Wilcoxon test. Statistical significance is indicated as follows: *p < 0.05, **p < 0.01, ***p < 0.001, and ****p < 0.0001.

To assess whether the lack of methylation sites in miRNA‐targeted genes contributes to the observed interplay, we compared global DNA methylation levels between miRNA‐targeted and non‐miRNA‐targeted genes. For ion transport genes, those regulated by both DNA methylation and miRNAs exhibited significantly lower methylation levels compared to genes regulated solely by DNA methylation at 0, 24, and 48 h. The DNA methylation levels of miRNA‐targeted and non‐targeted genes were 0.193 ± 0.053 and 0.227 ± 0.082 at 0 h, 0.206 ± 0.071 and 0.230 ± 0.088 at 24 h, and 0.193 ± 0.056 and 0.238 ± 0.092 at 48 h, respectively (Figure 4A). A similar pattern was observed in FAA metabolism and biogenesis genes, where the DNA methylation levels of co‐regulated genes were significantly lower than those regulated solely by DNA methylation at all time points, except at 24 h. The DNA methylation levels of miRNA‐targeted and non‐targeted genes were 0.168 ± 0.028 and 0.178 ± 0.027 at 0 h, 0.166 ± 0.027 and 0.178 ± 0.034 at 48 h, 0.166 ± 0.026 and 0.180 ± 0.035 at 96 h, 0.170 ± 0.028 and 0.181 ± 0.032 at 120 h, and 0.170 ± 0.030 and 0.174 ± 0.029 at 144 h, respectively (Figure 4B). Additionally, we compared the number of DNA methylation sites between miRNA‐targeted and non‐targeted genes. The results showed that miRNA‐targeted genes harbored significantly fewer methylation sites than genes not regulated by miRNAs in both ion transport and FAA metabolism and biogenesis pathways. Specifically, the numbers of DNA methylation sites in FAA metabolism‐related genes were 1.95 ± 1.36 for miRNA‐targeted genes and 2.14 ± 1.84 for non‐targeted genes, while for ion transport‐related genes, the corresponding values were 3.17 ± 3.13 and 3.67 ± 4.98, respectively (Figure 4C). This pattern supports that the absence of DNA methylation sites serves as a structural basis for the predominant regulation by miRNAs. However, the variation observed across different time points suggests that additional mechanisms contribute to the dynamic interplay between these two regulatory layers, particularly in genes with methylation‐capable sites that can be methylated in response to environmental stress.

Besides the functional interplay between miRNA regulation and DNA methylation modification, we found evidence in two genes indicating that these mechanisms also regulate each other. For example, several DEmiRNAs directly regulated the *Methyl‐CpG Binding Domain Protein 2 (MBD2)* gene, which plays a critical role in DNA methylation modification. Specifically, cin‐miR‐4046‐5p binds to the 3′UTR of *MBD2* and positively regulates its expression, while cin‐miR‐4059‐5p and cin‐miR‐92f‐5p bind to the CDS region of *MBD2*, exhibiting negative and positive regulation, respectively (Table S3). Similarly, one key gene involved in miRNA biogenesis is also influenced by DNA methylation. The *microprocessor complex subunit DiGeorge syndrome Critical Region 8 (DGCR8)* gene contains three DNA methylation sites within its gene body, and their methylation levels increased following recurrent salinity stress (Table S4).

### 3.4 Statistical Analysis of miRNA‐ and DNA Methylation‐Mediated Complementary Regulation

To assess whether the overlap between miRNA binding and DNA methylation within the same genes was non‐random, we conducted permutation tests by randomly sampling gene sets (1000 iterations) with the same numbers of miRNA‐regulated and DNA methylation‐regulated genes. The observed number of dually regulated genes was significantly higher than expected under the null distribution (*p* < 0.01; Figure S4), indicating that the co‐occurrence of the two mechanisms is not due to chance of events.

We next investigated whether dually regulated genes converged on specific stress‐related pathways. GO and KEGG enrichment analyses revealed no significant enrichment in canonical osmotic stress pathways (Table S5, FDR > 0.05). Instead, these genes were dispersed across diverse functional categories, suggesting that dual regulation should not target a narrow set of pathways but provide complementary regulation across multiple functions, likely supporting fine‐tuned and flexible stress responses.

Finally, we compared the magnitude of expression changes among three groups: (i) miRNA‐only regulated genes, (ii) methylation‐only regulated genes, and (iii) dually regulated genes. Wilcoxon tests showed no significant differences in |log_2_(fold change)| among the groups (*p* > 0.05; Figure S5). Thus, the dual regulation does not amplify gene expression changes but rather provides complementary modulation across distinct regulatory layers and genomic regions.

## 4 Discussion

Understanding the detailed mechanisms associated with response strategies is essential for explaining invasion success, particularly how invaders strategically employ interactive mechanisms such as miRNAs and DNA methylation. Upon exposure to repeated salinity stress, *C. robusta* primarily activated biological processes related to water and ion transport, FAA metabolism/biogenesis in a stress duration‐dependent pattern (Figure 1). Both miRNA‐mediated regulation and DNA methylation were involved in modulating the expression of functional genes above (Figure 1, Table 1), and individual miRNAs targeted multiple genes involved in similar functional pathways in a diverse regulatory pattern (Figure 2 and Figure S2). Moreover, these two epigenetic mechanisms exhibit functional coordination, targeting the same biological pathways but acting on complementary genes or different regions within the same genes (Figure 3 and Figure S3). Additionally, miRNA targeting reduced the global DNA methylation levels of genes, suggesting that reduced DNA methylation should serve as a potential structural basis for miRNA targeting (Figure 4 and Figure S3). However, dynamic DNA methylation levels suggest additional mechanisms, especially in genes with potential methylation sites. Collectively, our findings highlight the complementary roles of miRNAs and DNA methylation in regulating the expression of functional genes in response to environmental stress. These results offer novel insights into the molecular mechanisms underlying stress resilience in invasive species and underscore the critical importance of integrative epigenetic regulation in facilitating rapid adaptation to changing environments.

### 4.1 Osmolyte Shift Regulated by miRNAs During Stress

*C. robusta* recruited osmolytes (FAA and ions) to maintain osmotic homeostasis, and an osmolyte‐related gene shift occurred throughout the stress response, involving two noteworthy aspects. The first one pertains to the diversity of osmolytes. For example, Ca^2+^ transport‐related genes were regulated at 24 and 48 h and L‐cystine transport‐related genes at 144 h (Figure 2). The functional shift can be attributed to the limitations in osmolyte concentrations and the potential disruption of osmotic homeostasis by sustained ion transport. Under hypertonic conditions, cells recruit compatible organic osmolytes to counteract osmotic stress. Moreover, different species show preferences for specific osmolytes. In *C. robusta* , ions were identified as the preferential osmolytes (Yan et al. 2025), and a similar pattern was observed in *Erodona mactroides* (Medeiros et al. 2020). In contrast, FAAs were found as the preferential osmolytes in *C. savignyi* (Huang et al. 2023), while some species employed both FAAs and ions to cope with salinity fluctuations (e.g., Meng et al. 2013). However, the synthesis of these osmolytes is energetically costly and occurs gradually, making them suitable for fine‐tuning osmotic balance rather than serving as immediate responders to acute changes in salinity (Bai et al. 2019; Munns et al. 2020). Prolonged reliance on these osmolytes without concurrent ion transport may lead to osmotic imbalance, prompting the activation of alternative pathways to restore homeostasis (Burg and Ferraris 2008; Shabala and Shabala 2011). Additionally, the regulation of osmolyte transport is intricately linked to cell volume and ionic strength. For example, intracellular ionic strength can influence the activity of volume‐sensitive anion channels, which are involved in the release of organic osmolytes during volume regulation (Wittels et al. 2000). This suggests that sustained ion transport could affect the availability and regulation of osmolytes, further complicating the maintenance of osmotic balance.

The second aspect is the diversity of target genes for the same osmolytes. For example, tryptophan metabolism was adjusted at both 24 and 48 h, with different target genes regulated at each sampling point: *cyp2j2_1* at 24 h and *cyp2u1* at 48 h (Figure 2B). A similar shift in miRNA target genes has been observed in several species under recurrent high‐temperature stress, such as rice where miR‐167e specifically responded to the first stress and targeted *HSFA2* and *HSP70*, while miR‐1846d‐5p responded to the second stress and regulated *HSF* (Kushawaha et al. 2021). In addition, previous studies in animals have highlighted the critical regulatory roles of miRNAs under repeated stress (Uchida et al. 2008). However, there is no evidence of a transformation among functional genes with the same role at different stages, suggesting that the switchover phenomenon may be species‐specific. The sequential activation of genes during stress responses may allow organisms to adjust, repair damage, and maintain homeostasis under varying stress intensities and durations, ensuring that cellular functions are well maintained (Jovic et al. 2017; Todaka et al. 2017).

### 4.2 Diverse Binding Sites and Regulatory Effects Mediated by Both miRNA and DNA Methylation

The canonical mechanism of microRNA (miRNA) regulation involves binding to the 3′UTR of target mRNAs, resulting in translational repression or mRNA degradation (Dalmay 2013). However, increasing evidence suggests that miRNAs can also bind to alternative regions, including the 5′UTR and CDS, and in some cases, even promote gene expression (M. Pu et al. 2019; Xiao et al. 2017). In our study, we identified miRNA binding sites within all three regions including the 3′UTR, CDS, and 5′UTR, with the CDS emerging as the predominant target site (Figure S1A,B). This observation is consistent with our previous miRNAome‐wide analysis in this species (Yan et al. 2025), as well as findings in other organisms such as humans and green algae (Boudreau et al. 2014; Chung et al. 2017). Notably, both positive and negative regulatory effects were observed across all binding regions (Figure S1A,B), underscoring the complexity of miRNA‐mediated gene regulation, a pattern also seen in several taxa including tunicates (Yan et al. 2025) and even in viruses such as *Hepatitis C* (Moretti et al. 2010). Although the mechanisms underlying this regulatory diversity are not yet fully understood, several possible explanations have been proposed. For example, miRNA binding within the CDS often results in translational inhibition without significantly affecting mRNA stability, possibly due to interference with ribosome elongation (Hausser et al. 2013). Additionally, some miRNAs can simultaneously bind both the 5′UTR and 3′ UTR of a transcript which is termed “miBridge” interactions, often resulting in synergistic regulatory effects (Lytle et al. 2007). These diverse and sometimes conserved mechanisms across taxa emphasize the evolutionary importance and functional flexibility of miRNA‐mediated post‐transcriptional regulation in shaping gene expression and phenotypic diversity.

For DNA methylation, we also observed diverse regulatory patterns in both binding sites and their effects on gene expression (Table 1). DNA methylation can occur at both the promoter and gene body regions, with our study confirming this phenomenon and revealing that the gene body region contains a relatively higher proportion of methylation (Table 1). While the canonical role of promoter DNA methylation is to inhibit gene expression, examples of positive regulation have also been documented. For instance, hypermethylation of the *foxa2* promoter has been shown to promote its transcription (Halpern et al. 2014). This observed promotion of expression is likely linked to the release of repressive proteins, thereby facilitating the activation of *foxa2* expression (Halpern et al. 2014). Similarly, both positive and negative regulation were observed in this study when DNA methylation occurred in the promoter region, with the frequency of these two regulatory effects being similar. This pattern challenges the conventional view that promoter methylation mainly represses gene expression. Instead, it highlights a context‐dependent role of DNA methylation in gene regulation, where methylation can facilitate gene activation by disrupting repressive protein binding, or many other unknown mechanisms.

Furthermore, DNA methylation within the gene body was found to either inhibit or promote gene expression. For example, methylation of the *slc24a2* gene body was associated with expression repression, while methylation of the *slc10a2* gene body led to expression promotion (Table 1). Gene body methylation can exert both positive and negative regulatory effects, and the mechanisms underlying expression promotion may involve blocking intragenic promoters or influencing the activity of repetitive DNA sequences within the transcriptional unit (Maunakea et al. 2010). Despite these insights, the precise molecular mechanisms responsible for expression inhibition remain unclear, and further research is needed to understand the diverse roles of gene body methylation in transcriptional regulation.

### 4.3 Functional Complementation Between miRNA‐ and Methylation‐Based Regulation

Interestingly, DNA methylation and miRNA regulation affected distinct sets of genes and biological functions (Figure 3), suggesting their complementary roles in the rapid response to salinity stress. This pattern of relatively independent regulatory effects has also been observed under thermal stress (Ren et al. 2024), in the regulation of psoriasis (Laha et al. 2023), and during *Mycobacterium tuberculosis* infection (Looney et al. 2021). One proposed mechanism underlying this complementarity is the difference in their binding sites: DNA methylation typically occurs in promoter regions, whereas miRNAs generally bind to 3′ UTRs of target mRNAs. However, miRNAs can also bind to other regions, and DNA methylation may occur within gene bodies, indicating that spatial separation of binding sites may not fully explain their complementary regulation. An alternative explanation is that these two mechanisms act at different stages of gene expression: DNA methylation functions primarily at the pre‐transcriptional level, while miRNAs exert their effects post‐transcriptionally. Nevertheless, the precise temporal and mechanistic interactions between these pathways require further investigation (Su et al. 2011a). In addition, other regulatory mechanisms such as alternative splicing (AS) and alternative polyadenylation (APA) were also observed to affect distinct gene sets during the rapid salinity response in *Ciona* ascidians (Huang et al. 2023). All these findings suggest that the observed independence among regulatory mechanisms may arise from their reliance on distinct regulatory machineries involving different *cis*‐elements and *trans*‐acting factors (Huang et al. 2023; Sadek et al. 2019; Schaefke et al. 2018). Interestingly, we also found that miRNAs and DNA methylation regulate their respective functional genes (Tables S3 and S4). Previous studies have shown that miRNAs can regulate DNA methylation‐related genes, including *DNMT* and *MBD2*, thereby influencing global DNA methylation levels (Wada et al. 2010; Wu et al. 2016). Conversely, DNA methylation of miRNA biogenesis genes such as *Dicer* and *Drosha* may affect overall miRNA abundance (Heydarzadeh et al. 2021). In our study, however, we found no direct evidence that DNA methylation influences miRNA expression levels. Nonetheless, the direct crosstalk between these two key epigenetic mechanisms may represent an additional layer contributing to their functional complementarity. Collectively, our findings support that the interplay between DNA methylation and miRNA regulation contributes to the regulatory complexity observed in vertebrate organisms and diverse cell types, potentially offering new insights into the epigenetic forces driving the evolution of biological complexity (Gu et al. 2009; Mandrioli 2007).

As for the genes that were affected by DNA methylation and miRNA simultaneously, the binding sites of DNA methylation and miRNA on a single gene were often spatially divergent (Figure S3). Although research focusing specifically on the spatial interplay between miRNA binding and DNA methylation remains limited, a few studies have reported analogous regulatory patterns. For example, in skeletal muscle, *eIF5A1* expression is co‐regulated by miR‐434‐3p, which binds 3′ UTR, and DNA methylation at 5′ UTR (Shang et al. 2016). When both mechanisms target the same gene, albeit at distinct regions, they may fine‐tune gene expression through a multilayered regulatory network. For instance, studies on oncogenes and tumor suppressor genes have identified cases where promoter hypermethylation silences gene transcription, while miRNAs simultaneously downregulate residual mRNA expression, suggesting a cooperative suppression mechanism (Li et al. 2018).

Moreover, during the rapid osmotic stress response in *C. robusta* , miRNA regulation influenced a greater number of genes than DNA methylation (Figure 3A,B). A similar phenomenon was observed in primary human macrophages following *M. tuberculosis* infection (Looney et al. 2021). The broad scope of miRNA targets could offer a potential explanation, as over one‐third of protein‐coding genes in the human genome are targeted by miRNAs (Friedman et al. 2009). Previous studies in *C. robusta* have also demonstrated that nearly 25% of its genes are regulated by miRNAs (Yan et al. 2025). Interestingly, a multi‐omics analysis of thermal stress in fish revealed a contrasting pattern: while DNA methylation showed significant changes, no differentially expressed miRNAs were detected, suggesting that DNA methylation may play a more prominent role than miRNAs in the thermal stress response (Ren et al. 2024). Thus, the relative contributions of miRNA regulation and DNA methylation to stress responses are species‐specific and may be context‐dependent. This underscores the need for further investigation into their interplay and temporal dynamics, particularly in relation to other epigenetic modifications, to better understand their roles in phenotypic plasticity and adaptive responses to environmental challenges.

Our additional statistical analyses provide new insights into the proposed “complementary regulation” mechanism between miRNA and DNA methylation. The permutation test clearly demonstrates that the co‐occurrence of these two mechanisms in the same genes is significantly higher than expected by chance, confirming a non‐random structural relationship (Figure S4). However, contrary to the expectation of functional convergence, dually regulated genes were not significantly enriched in specific stress‐responsive pathways (Table S5). Instead, they were distributed across diverse biological processes, suggesting that complementary regulation should operate in a multilayered fashion. Rather than targeting a single pathway, the interplay between miRNA and methylation likely ensures regulatory robustness and flexibility across multiple functions. Furthermore, interaction analysis revealed that dual regulation does not produce stronger additive effects on gene expression compared to single regulation (Figure S5), indicating that its role lies in fine‐tuning gene regulation across different genomic and regulatory layers rather than amplifying expression changes. Such complementary regulation may be particularly advantageous under environmental stress, enabling organisms to balance stability and plasticity in their responses. Overall, while the co‐occurrence of miRNA binding and DNA methylation is clearly non‐random, their roles appear complementary rather than convergent or additive, highlighting the importance of considering multi‐layered regulation when interpreting rapid genomic responses.

As critical epigenetic mechanisms, miRNA and DNA methylation mediate rapid responses at the gene expression level, not only facilitating phenotypic plasticity but also promoting the long‐term adaptation of invasive species, thereby contributing to invasion success. By enabling individuals to tolerate a wider range of environmental conditions and expand their ecological niche, miRNA and DNA methylation enhance invasion capacity. This process occurs much more rapidly than genetic variation, helping invaders cross barriers during the early stages of invasions and further establish in new habitats (Xiang et al. 2023). Regarding long‐term adaptation, the 'plasticity‐first' hypothesis, although debated, proposes that repeated stress can stabilize specific epigenetic patterns to preserve adaptive phenotypes over extended periods (Levis and Pfennig 2016). Therefore, it is crucial to assess the contribution of epigenetic mechanisms, such as miRNA and DNA methylation, in enhancing both invasion capacity and long‐term adaptation.

In conclusion, this study observed rapid responses in water transport, FAA metabolism/biogenesis, and ion transport processes during repeated osmotic stress in *C. robusta* . Two critical epigenetic mechanisms, miRNA regulation and DNA methylation, were found to participate in the regulation of functional genes, and evidence suggests that these two layers of regulation interact to fine‐tune stress responses. The coordinated involvement of these mechanisms highlights the complexity of gene expression control under environmental challenges and points toward a potential epigenetic memory that could facilitate acclimation during recurrent osmotic stress events. Nevertheless, it is important to emphasize the correlative nature of the present findings, as omics‐based associations cannot fully establish causation. Future investigations should experimentally validate the expression and functional roles of key miRNAs, their predicted mRNA targets, and DNA methylation changes through integrative approaches such as qRT‐PCR, bisulfite sequencing, and reporter assays. Moreover, functional knockdown or overexpression of candidate miRNAs, combined with methylation editing technologies, would provide deeper insights into the causative roles of these mechanisms. Such studies will not only help verify the proposed regulatory interactions but also enhance our understanding of how epigenetic regulation contributes to the adaptive capacity.

## Disclosure

Declaration of AI Use: We have not used AI‐assisted technologies in creating this article.

## Ethics Statement

The authors have nothing to report.

## Conflicts of Interest

The authors declare no conflicts of interest.

## Supporting information


**Figure S1:** Schematic illustration of the recurrent salinity stress experiment. After acclimation, healthy *Ciona robusta* individuals were randomly selected for the stress treatments. In the first round, individuals were exposed to high salinity (40‰) for 72 h (S1 stage) and sampled at 0, 24, and 48 h. They were then returned to ambient salinity (30‰) for 24 h (Recovery, R stage) and sampled at 96 h. Finally, the individuals underwent a second round of high‐salinity stress (40‰, S2 stage) and were sampled at 120 and 144 h.
**Figure S2:** Genome distribution and regulatory correlations of miRNA regulation. Panels (A) and (B) depict regulatory relationships for ion transport and Free Amino Acid (FAA) metabolism, respectively. Positive and negative correlations are indicated in red and green, while the Coding DNA Sequence (CDS), 5′ Untranslated Region (5′ UTR), and 3′ Untranslated Region (3′ UTR) regions are shown in purple, yellow, and blue.
**Figure S3:** Examples of miRNA binding sites and DNA methylation sites on target genes co‐regulated by both mechanisms. Panels (A) and (B) show functional genes involved in ion transport and Free Amino Acid (FAA) metabolism and biogenesis, respectively.
**Figure S4:** Frequency distribution of randomly expected dual‐regulated genes based on 1000 permutation tests. The null distribution (blue bars) was generated by randomly sampling gene sets of the same size as the actual miRNA‐targeted and methylated gene sets, and the dashed vertical line indicates the mean of the empirical null distribution.
**Figure S5:** Global |log_2_(fold change)| of miRNA‐regulated, DNA methylation‐regulated, and dual‐regulated genes at different sampling points. Panels (A)‐(E) correspond to 24, 48, 96, 120, and 144 h, respectively. Gene categories are color‐coded as follows: miRNA‐regulated (blue), DNA methylation‐regulated (red), and dual‐regulated (green).
**Table S1:** Functional genes related to three canonical strategies.
**Table S2:** The miRNA‐target pair related to ion transport and free amino acid (FAA) metabolism and biogenesis.
**Table S3:** The DNA methylation gene that regulated by miRNAs.
**Table S4:** The key gene during miRNA biogenesis and decay processes influenced by DNA methylation.
**Table S5:** The GO terms enriched by dual‐regulated genes.

## Data Availability

All raw high‐throughput mRNA and DNA methylation sequencing data were deposited in the National Center for Biotechnology Information (NCBI) Sequence Read Archive (SRA) under accession number PRJNA775866, and the raw miRNA‐seq data was deposited in the SRA database under the number PRJNA1120970.
