# Supplementary material for: Complementary Roles of DNA Methylation and miRNA in Regulating Gene Expression Under Environmental Stress in Biological Invasions
Source: Evol Appl. 2025 Nov 8;18(11):e70178. doi: 10.1111/eva.70178 (PMC12595603; doi:10.1111/eva.70178)
Supplement: Supplementary file 1 — Figure S1: Schematic illustration of the recurrent salinity stress experiment. After acclimation, healthy Ciona robusta individuals were randomly selected for the stress treatments. In the first round, individuals were exposed to high salinity (40‰) for 72 h (S1 stage) and sampled at 0, 24, and 48 h. They were then returned to ambient salinity (30‰) for 24 h (Recovery, R stage) and sampled at 96 h. Finally, the individuals underwent a second round of high‐salinity stress (40‰, S2 stage) and were sampled at 120 and 144 h. Figure S2: Genome distribution and regulatory correlations of miRNA regulation. Panels (A) and (B) depict regulatory relationships for ion transport and Free Amino Acid (FAA) metabolism, respectively. Positive and negative correlations are indicated in red and green, while the Coding DNA Sequence (CDS), 5′ Untranslated Region (5′ UTR), and 3′ Untranslated Region (3′ UTR) regions are shown in purple, yellow, and blue. Figure S3: Examples of miRNA binding sites and DNA methylation sites on target genes co‐regulated by both mechanisms. Panels (A) and (B) show functional genes involved in ion transport and Free Amino Acid (FAA) metabolism and biogenesis, respectively. Figure S4: Frequency distribution of randomly expected dual‐regulated genes based on 1000 permutation tests. The null distribution (blue bars) was generated by randomly sampling gene sets of the same size as the actual miRNA‐targeted and methylated gene sets, and the dashed vertical line indicates the mean of the empirical null distribution. Figure S5: Global |log2(fold change)| of miRNA‐regulated, DNA methylation‐regulated, and dual‐regulated genes at different sampling points. Panels (A)‐(E) correspond to 24, 48, 96, 120, and 144 h, respectively. Gene categories are color‐coded as follows: miRNA‐regulated (blue), DNA methylation‐regulated (red), and dual‐regulated (green). Table S1: Functional genes related to three canonical strategies. Table S2: The miRNA‐target pair related to ion tra [file EVA-18-e70178-s001.docx]

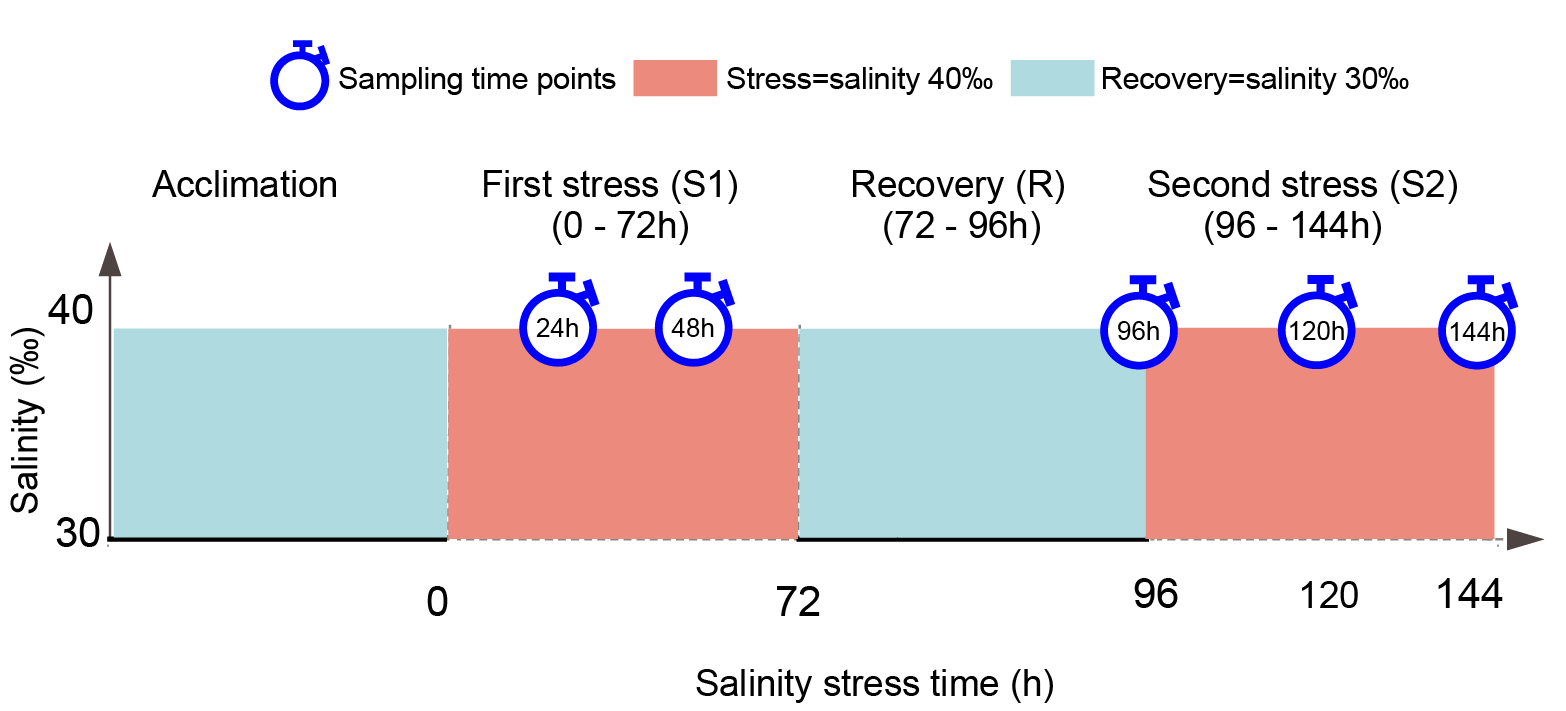


**Figure S1.** Schematic illustration of the recurrent salinity stress experiment. After acclimation, healthy *Ciona robusta* individuals were randomly selected for the stress treatments. In the first round, individuals were exposed to high salinity (40‰) for 72 h (S1 stage) and sampled at 0, 24, and 48 h. They were then returned to ambient salinity (30‰) for 24 h (Recovery, R stage) and sampled at 96 h. Finally, the individuals underwent a second round of high-salinity stress (40‰, S2 stage) and were sampled at 120 and 144 h.


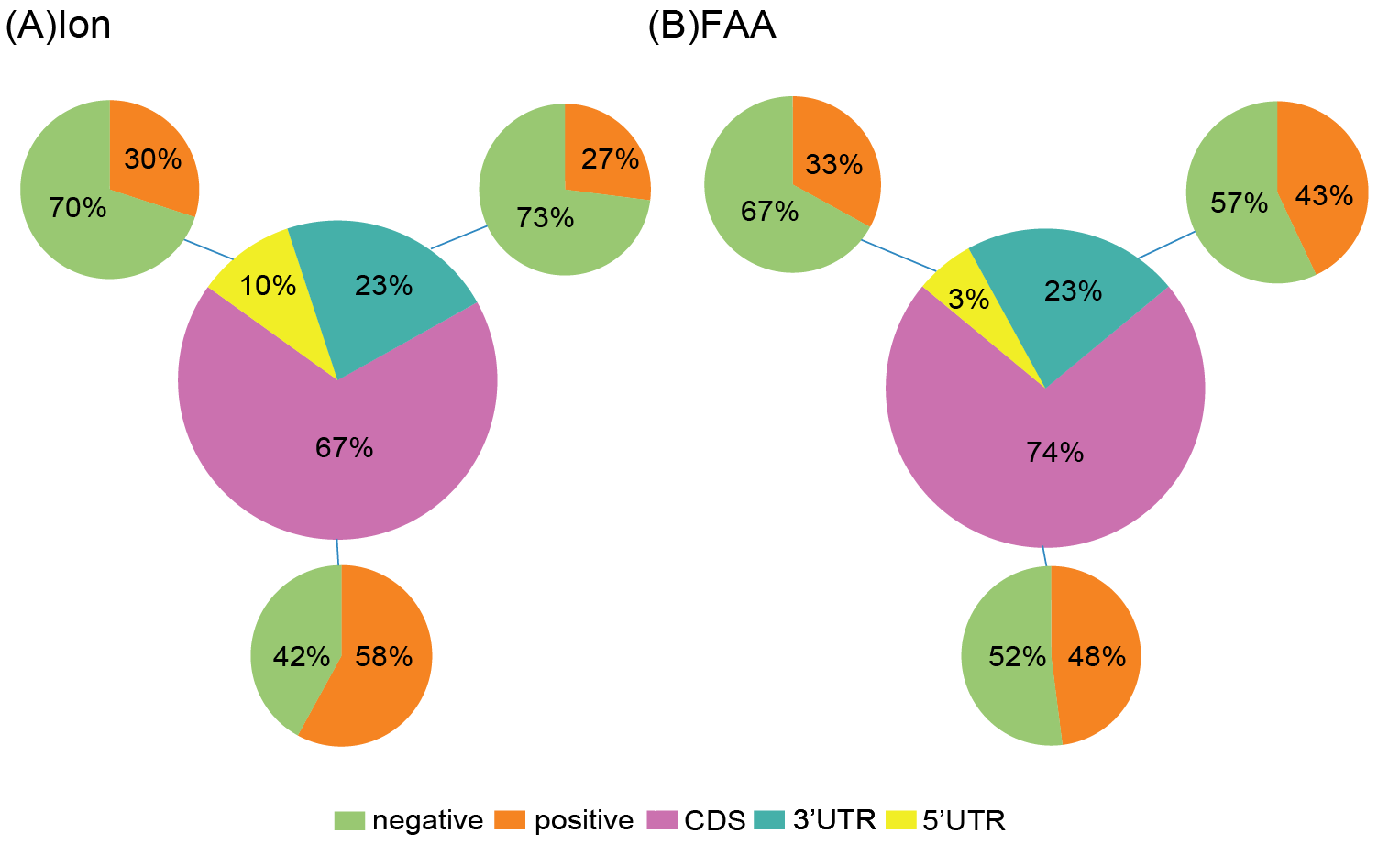


**Figure S2** Genome distribution and regulatory correlations of miRNA regulation. Panels (A) and (B) depict regulatory relationships for ion transport and Free Amino Acid (FAA) metabolism, respectively. Positive and negative correlations are indicated in red and green, while the Coding DNA Sequence (CDS), 5' Untranslated Region (5′ UTR), and 3' Untranslated Region (3′ UTR) regions are shown in purple, yellow, and blue.


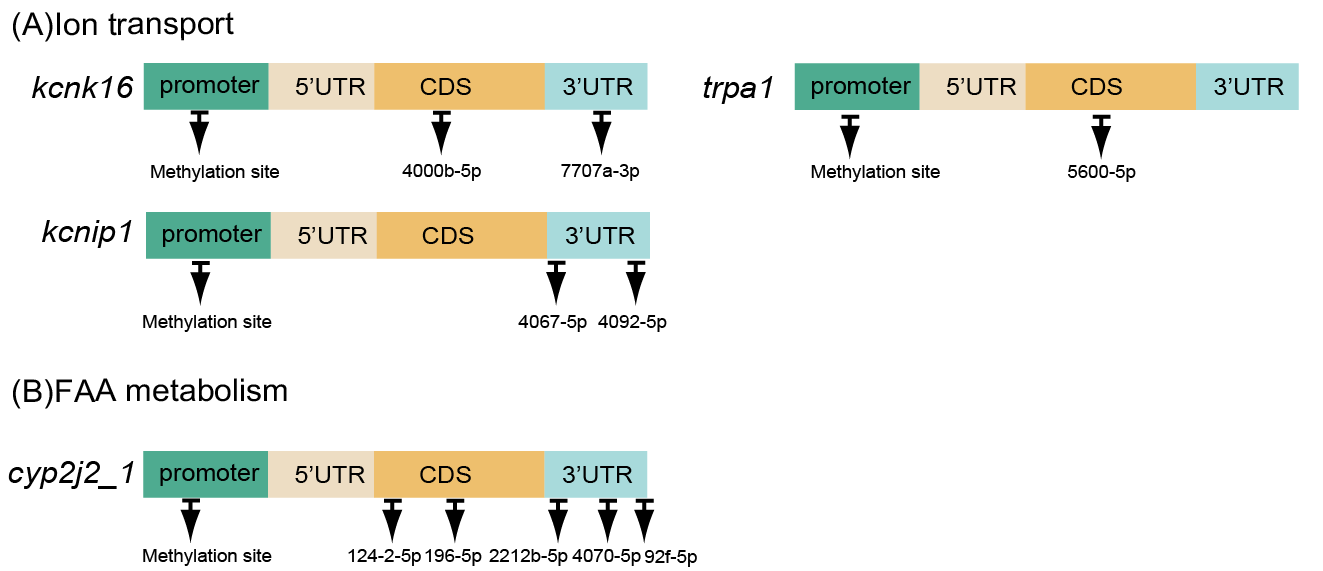


**Figure S3** Examples of miRNA binding sites and DNA methylation sites on target genes co-regulated by both mechanisms. Panels (A) and (B) show functional genes involved in ion transport and Free Amino Acid (FAA) metabolism and biogenesis, respectively.


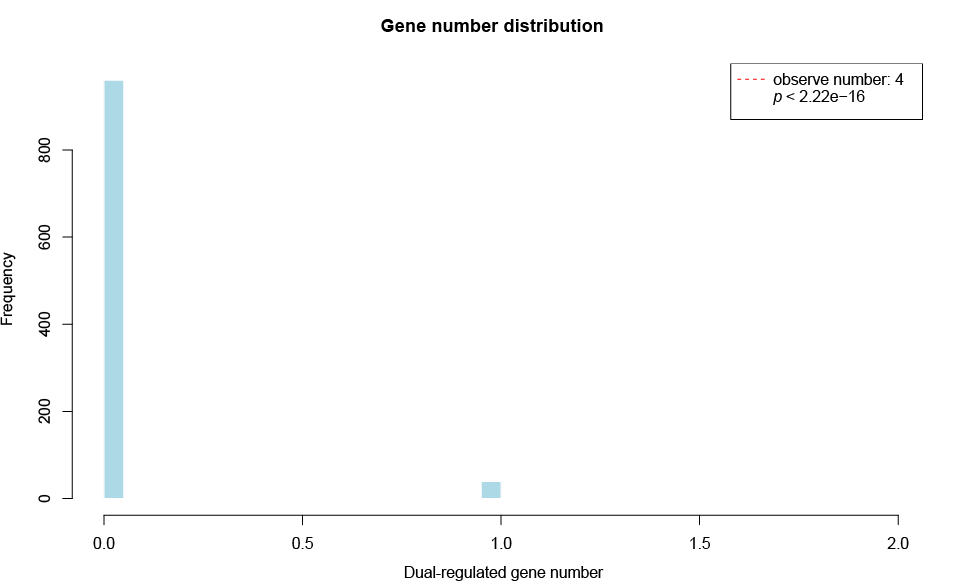


**Figure S4** Frequency distribution of randomly expected dual-regulated genes based on 1,000 permutation tests. The null distribution (blue bars) was generated by randomly sampling gene sets of the same size as the actual miRNA-targeted and methylated gene sets, and the dashed vertical line indicates the mean of the empirical null distribution.


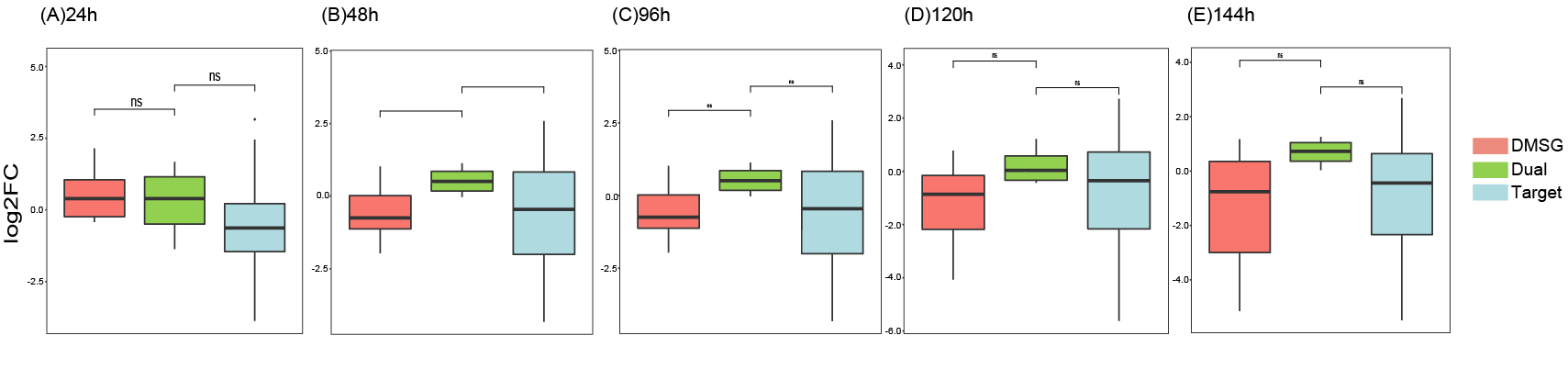


**Figure S5** Global |log₂(fold change)| of miRNA-regulated, DNA methylation-regulated, and dual-regulated genes at different sampling points. Panels (A)-(E) correspond to 24, 48, 96, 120, and 144 h, respectively. Gene categories are color-coded as follows: miRNA-regulated (blue), DNA methylation-regulated (red), and dual-regulated (green).

**Table S1** Functional genes related to three canonical strategies

| No. | Gene ID | Gene name | Category | Protein name |
| --- | --- | --- | --- | --- |
| 1 | KY. Chr12.726 | AQP8_1 | water | aquaporin 8 |
| 2 | KY. Chr8.934 | AQP8_3 | water | aquaporin 8 |
| 3 | KY. Chr9.915 | AQP3_2 | water | aquaporin 3 |
| 4 | KY. Chr10.111 | ATP1A3_2 | ion | ATPase Na+/K+ transporting subunit alpha 3 |
| 5 | KY. Chr11.396 | ATP1A4_3 | ion | ATPase Na+/K+ transporting subunit alpha 4 |
| 6 | KY. Chr10.992 | SLC8A1_1 | ion | solute carrier family 8 member 1 |
| 7 | KY. Chr7.932 | SLC8A1_2 | ion | solute carrier family 8 member 1 |
| 8 | KY. Chr4.1141 | SLC8A3_1 | ion | solute carrier family 8 member 3 |
| 9 | KY. Chr8.1128 | SLC8A3_2 | ion | solute carrier family 8 member 3 |
| 10 | KY. Chr8.473 | SLC10A1 | ion | solute carrier family 10 member 1 |
| 11 | KY. Chr9.635 | SLC10A2_2 | ion | solute carrier family 10 member 2 |
| 12 | KY. Chr9.637 | SLC10A2_3 | ion | solute carrier family 10 member 2 |
| 13 | KY. Chr7.1134 | SLC10A3 | ion | solute carrier family 10 member 3 |
| 14 | KY. Chr3.745 | SLC10A5 | ion | solute carrier family 10 member 5 |
| 15 | KY. Chr3.453 | SLC10A6 | ion | solute carrier family 10 member 6 |
| 16 | KY. Chr2.1411 | SLC10A7 | ion | solute carrier family 10 member 7 |
| 17 | KY. Chr5.310 | SLC12A3_1 | ion | solute carrier family 12 member 3 |
| 18 | KY. Chr5.311 | SLC12A3_2 | ion | solute carrier family 12 member 3 |
| 19 | KY. Chr3.71 | SLC12A4 | ion | solute carrier family 12 member 4 |
| 20 | KY. Chr4.1016 | SLC12A5 | ion | solute carrier family 12 member 5 |
| 21 | KY. Chr4.1205 | SLC12A9 | ion | solute carrier family 12 member 9 |
| 22 | KY. Chr11.289 | SLC13A2 | ion | solute carrier family 13 member 2 |
| 23 | KY. Chr2.831 | SLC13A3_1 | ion | solute carrier family 13 member 3 |
| 24 | KY. Chr3.1566 | SLC13A4 | ion | solute carrier family 13 member 4 |
| 25 | KY. Chr11.395 | SLC13A5 | ion | solute carrier family 13 member 5 |
| 26 | KY. Chr11.853 | SLC20A1_1 | ion | solute carrier family 20 member 1 |
| 27 | KY. Chr3.1314 | SLC20A1_2 | ion | solute carrier family 20 member 2 |
| 28 | KY. Chr5.969 | SLC20A1_3 | ion | solute carrier family 20 member 3 |
| 29 | KY. Chr4.678 | SLC20A1_5 | ion | solute carrier family 20 member 5 |
| 30 | KY. Chr4.682 | SLC20A1_6 | ion | solute carrier family 20 member 6 |
| 31 | KY. Chr3.433 | SLC23A2_1 | ion | solute carrier family 23 member 2 |
| 32 | KY. Chr3.65 | SLC23A2_2 | ion | solute carrier family 23 member 2 |
| 33 | KY. Chr9.624 | SLC24A2 | ion | solute carrier family 24 member 2 |
| 34 | KY. Chr8.189 | SLC24A5 | ion | solute carrier family 24 member 5 |
| 35 | KY. Chr12.566 | SLC28A1 | ion | solute carrier family 28 member 1 |
| 36 | KY. Chr3.114 | SLC28A3 | ion | solute carrier family 28 member 3 |
| 37 | KY. Chr1.2378 | KCNA1 | ion | potassium voltage-gated channel subfamily A member 1 |
| 38 | KY. Chr1.507 | KCNAB2 | ion | potassium voltage-gated channel subfamily A regulatory beta subunit 2 |
| 39 | KY. Chr1.595 | KCND2_1 | ion | potassium voltage-gated channel subfamily D member 2 |
| 40 | KY. Chr10.126 | KCNJ1 | ion | potassium voltage-gated channel subfamily J member 1 |
| 41 | KY. Chr10.620 | KCNQ5 | ion | potassium voltage-gated channel subfamily Q member 5 |
| 42 | KY. Chr11.1161 | KCND2_2 | ion | potassium voltage-gated channel subfamily D member 2 |
| 43 | KY. Chr11.479 | KCNJ5 | ion | potassium voltage-gated channel subfamily J member 5 |
| 44 | KY. Chr11.487 | KCNE5 | ion | potassium voltage-gated channel subfamily E member 5 |
| 45 | KY. Chr12.219 | KCNA4 | ion | potassium voltage-gated channel subfamily A member 4 |
| 46 | KY. Chr12.751 | KCNA3 | ion | potassium voltage-gated channel subfamily A member 3 |
| 47 | KY. Chr13.423 | KCNH3 | ion | potassium voltage-gated channel subfamily A member 3 |
| 48 | KY. Chr2.1147 | KCNS2 | ion | potassium voltage-gated channel subfamily S member 2 |
| 49 | KY. Chr2.947 | KCNV1_1 | ion | potassium voltage-gated channel subfamily V member 1 |
| 50 | KY. Chr3.337 | KCNV1_2 | ion | potassium voltage-gated channel subfamily V member 1 |
| 51 | KY. Chr3.738 | KCNIP4_1 | ion | potassium voltage-gated channel interacting protein 4 |
| 52 | KY. Chr4.1014 | KCNIP1 | ion | potassium voltage-gated channel interacting protein 1 |
| 53 | KY. Chr4.744 | KCNJ16 | ion | potassium voltage-gated channel subfamily J member 16 |
| 54 | KY. Chr4.773 | KCNJ12 | ion | potassium voltage-gated channel subfamily J member 12 |
| 55 | KY. Chr8.1033 | KCNIP4_2 | ion | potassium voltage-gated channel interacting protein 4 |
| 56 | KY. Chr8.1058 | KCNH5 | ion | potassium voltage-gated channel subfamily H member 5 |
| 57 | KY. Chr9.1118 | KCNS1 | ion | potassium voltage-gated channel modifier subfamily S member 1 |
| 58 | KY. UAContig6.39 | KCNQ3 | ion | potassium voltage-gated channel subfamily Q member 3 |
| 59 | KY. UAContig6.54 | KCNQ2_1 | ion | potassium voltage-gated channel subfamily Q member 2 |
| 60 | KY. UAContig7.18 | KCNQ2_2 | ion | potassium voltage-gated channel subfamily Q member 2 |
| 61 | KY. UAContig13.17 | KCNQ2_3 | ion | potassium voltage-gated channel subfamily Q member 2 |
| 62 | KY. UAContig51.14 | KCNQ2_4 | ion | potassium voltage-gated channel subfamily Q member 2 |
| 63 | KY. UAContig51.22 | KCNQ2_5 | ion | potassium voltage-gated channel subfamily Q member 2 |
| 64 | KY. UAContig47.3 | KCNQ2_6 | ion | potassium voltage-gated channel subfamily Q member 2 |
| 65 | KY. UAContig53.12 | KCNQ2_7 | ion | potassium voltage-gated channel subfamily Q member 2 |
| 66 | KY. UAContig49.20 | KCNQ2_8 | ion | potassium voltage-gated channel subfamily Q member 2 |
| 67 | KY. UAContig49.3 | KCNQ2_9 | ion | potassium voltage-gated channel subfamily Q member 2 |
| 68 | KY. UAContig32.3 | KCNH4 | ion | potassium voltage-gated channel subfamily H member 4 |
| 69 | KY. Chr1.2219 | CLCNKA | ion | chloride voltage-gated channel Ka |
| 70 | KY. Chr3.1376 | CLCN6_1 | ion | chloride voltage-gated channel 6 |
| 71 | KY. Chr7.685 | CLCN6_2 | ion | chloride voltage-gated channel 6 |
| 72 | KY. Chr7.91 | CLCN5_2 | ion | chloride voltage-gated channel 5 |
| 73 | KY. Chr8.1268 | CLCN2 | ion | chloride voltage-gated channel 2 |
| 74 | KY. Chr3.51 | CLIC5 | ion | chloride intracellular channel 5 |
| 75 | KY. Chr12.717 | SCN1A | ion | sodium voltage-gated channel alpha subunit 1 |
| 76 | KY. Chr3.1628 | SCN2A | ion | sodium voltage-gated channel alpha subunit 2 |
| 77 | KY. Chr4.815 | SCN4A_1 | ion | sodium voltage-gated channel alpha subunit 4 |
| 78 | KY. Chr14.215 | SCN4A_2 | ion | sodium voltage-gated channel alpha subunit 4 |
| 79 | KY. Chr1.526 | SCN5A_1 | ion | sodium voltage-gated channel alpha subunit 5 |
| 80 | KY. Chr1.567 | SCN5A_2 | ion | sodium voltage-gated channel alpha subunit 5 |
| 81 | KY. Chr1.931 | SCN5A_3 | ion | sodium voltage-gated channel alpha subunit 5 |
| 82 | KY. Chr11.378 | SCN5A_4 | ion | sodium voltage-gated channel alpha subunit 5 |
| 83 | KY. Chr4.605 | SCN7A_1 | ion | sodium voltage-gated channel alpha subunit 7 |
| 84 | KY. Chr14.953 | SCN7A_2 | ion | sodium voltage-gated channel alpha subunit 7 |
| 85 | KY. Chr3.1626 | SCN9A_1 | ion | sodium voltage-gated channel alpha subunit 9 |
| 86 | KY. UAContig47.1 | SCN9A_3 | ion | sodium voltage-gated channel alpha subunit 9 |
| 87 | KY. Chr1.899 | SCN9A_4 | ion | sodium voltage-gated channel alpha subunit 9 |
| 88 | KY. Chr5.634 | SCN11A | ion | sodium voltage-gated channel alpha subunit 11 |
| 89 | KY. Chr10.585 | CACNG3 | ion | calcium voltage-gated channel auxiliary subunit gamma 3 |
| 90 | KY. Chr10.927 | KCNK10_1 | ion | potassium two pore domain channel subfamily K member 10 |
| 91 | KY. Chr14.1120 | CACNA1H | ion | calcium voltage-gated channel subunit alpha1 H |
| 92 | KY. Chr5.968 | KCNK16 | ion | potassium two pore domain channel subfamily K member 16 |
| 93 | KY. Chr14.1175 | CACNA1I | ion | calcium voltage-gated channel subunit alpha1 I |
| 94 | KY. Chr1.2098 | KCNK18 | ion | potassium two pore domain channel subfamily K member 18 |
| 95 | KY. Chr1.2090 | HCN1 | ion | hyperpolarization activated cyclic nucleotide gated potassium channel 1 |
| 96 | KY. Chr9.206 | CACNA1A | ion | calcium voltage-gated channel subunit alpha1 A |
| 97 | KY. Chr2.2019 | TRPA1 | ion | transient receptor potential cation channel subfamily A member 1 |
| 98 | KY. Chr11.899 | CACNA1E | ion | calcium voltage-gated channel subunit alpha1 E |
| 99 | KY. Chr2.2125 | SLC9B1P1 | ion | solute carrier family 9 member B1 pseudogene 1 |
| 100 | KY. Chr3.139 | SLC3A1 | faa | solute carrier family 3 member 1 |
| 101 | KY. Chr14.621 | SLC7A1_1 | faa | solute carrier family 7 member 1 |
| 102 | KY. Chr2.208 | SLC7A1_2 | faa | solute carrier family 7 member 1 |
| 103 | KY. Chr3.1171 | SLC7A11_1 | faa | solute carrier family 7 member 11 |
| 104 | KY. Chr5.352 | SLC7A11_2 | faa | solute carrier family 7 member 11 |
| 105 | KY. Chr6.683 | SLC7A11_3 | faa | solute carrier family 7 member 11 |
| 106 | KY. Chr8.627 | SLC7A11_4 | faa | solute carrier family 7 member 11 |
| 107 | KY. Chr9.936 | SLC7A11_5 | faa | solute carrier family 7 member 11 |
| 108 | KY. UAContig5.5 | SLC7A11_6 | faa | solute carrier family 7 member 11 |
| 109 | KY. Chr9.982 | SLC7A13 | faa | solute carrier family 7 member 13 |
| 110 | KY. Chr2.1193 | SLC7A4_1 | faa | solute carrier family 7 member 4 |
| 111 | KY. Chr3.20 | SLC7A5 | faa | solute carrier family 7 member 5 |
| 112 | KY. Chr1.1339 | SLC7A6_1 | faa | solute carrier family 7 member 6 |
| 113 | KY. Chr1.1340 | SLC7A6_2 | faa | solute carrier family 7 member 6 |
| 114 | KY. Chr10.1159 | SLC7A8_1 | faa | solute carrier family 7 member 8 |
| 115 | KY. Chr11.280 | SLC7A8_2 | faa | solute carrier family 7 member 8 |
| 116 | KY. Chr1.1636 | SLC7A9_1 | faa | solute carrier family 7 member 9 |
| 117 | KY. Chr1.1643 | SLC7A9_2 | faa | solute carrier family 7 member 9 |
| 118 | KY. Chr1.2384 | SLC7A9_3 | faa | solute carrier family 7 member 9 |
| 119 | KY. Chr2.2175 | SLC7A9_4 | faa | solute carrier family 7 member 9 |
| 120 | KY. Chr2.789 | SLC7A9_5 | faa | solute carrier family 7 member 9 |
| 121 | KY. Chr3.1584 | SLC7A9_6 | faa | solute carrier family 7 member 9 |
| 122 | KY. Chr3.679 | SLC7A9_7 | faa | solute carrier family 7 member 9 |
| 123 | KY. Chr3.78 | SLC7A9_8 | faa | solute carrier family 7 member 9 |
| 124 | KY. Chr4.1106 | SLC7A9_9 | faa | solute carrier family 7 member 9 |
| 125 | KY. Chr11.852 | SLC17A5_1 | faa | solute carrier family 17 member 5 |
| 126 | KY. Chr11.943 | SLC17A5_2 | faa | solute carrier family 17 member 5 |
| 127 | KY. Chr11.944 | SLC17A5_3 | faa | solute carrier family 17 member 5 |
| 128 | KY. Chr11.945 | SLC17A5_4 | faa | solute carrier family 17 member 5 |
| 129 | KY. Chr12.341 | SLC17A5_5 | faa | solute carrier family 17 member 5 |
| 130 | KY. Chr4.1012 | SLC17A5_6 | faa | solute carrier family 17 member 5 |
| 131 | KY. Chr12.868 | CYP1A2_1 | faa | cytochrome P450 family 1 subfamily A member 2 |
| 132 | KY. Chr2.2263 | CYP1A1_1 | faa | cytochrome P450 family 1 subfamily A member 1 |
| 133 | KY. Chr2.420 | CYP11A1_1 | faa | cytochrome P450 family 11 subfamily A member 1 |
| 134 | KY. Chr2.421 | CYP11A1_2 | faa | cytochrome P450 family 11 subfamily A member 1 |
| 135 | KY. Chr5.202 | CYP1B1_1 | faa | cytochrome P450 family 1 subfamily B member 1 |
| 136 | KY. Chr8.880 | CYP1B1_2 | faa | cytochrome P450 family 1 subfamily B member 1 |
| 137 | KY. Chr8.177 | CYP2J2_1 | faa | cytochrome P450 family 2 subfamily J member 2 |
| 138 | KY. Chr2.446 | CYP27B1 | faa | cytochrome P450 family 27 subfamily B member 1 |
| 139 | KY. Chr12.461 | CYP4F2_1 | faa | cytochrome P450 family 4 subfamily F member 2 |
| 140 | KY. Chr9.138 | CYP2J2_2 | faa | cytochrome P450 family 2 subfamily J member 2 |
| 141 | KY. Chr5.142 | CYP2U1 | faa | cytochrome P450 family 2 subfamily U member 1 |
| 142 | KY. Chr11.1260 | CYP2D6 | faa | cytochrome P450 family 2 subfamily D member 6 |
| 143 | KY. Chr12.623 | CYP4F2_2 | faa | cytochrome P450 family 4 subfamily F member 2 |
| 144 | KY. Chr7.1067 | CYP7A1 | faa | cytochrome P450 family 7 subfamily A member 1 |
| 145 | KY. Chr1.2349 | SRR | faa | serine racemase |
| 146 | KY. Chr11.281 | GAPDH | faa | glyceraldehyde-3-phosphate dehydrogenase |
| 147 | KY. Chr5.665 | PGAM2_2 | faa | phosphoglycerate mutase 2 |
| 148 | KY. Chr5.53 | PC_1 | faa | pyruvate carboxylase |

**Table S2** The miRNA-target pair related to ion transport and free amino acid (FAA) metabolism and biogenesis

| abbreviation | miRNA | target | function |
| --- | --- | --- | --- |
| L1 | cin-miR-4059-5p | SLC7A13 | L-cystine transport |
| L2 | cin-miR-4037-5p | SLC7A6 | L-cystine transport |
| L3 | cin-miR-4067-5p | SLC7A6 | L-cystine transport |
| L4 | cin-miR-4070-5p | SLC7A6 | L-cystine transport |
| L5 | cin-miR-4063-5p | SLC7A8 | L-cystine transport |
| L6 | cin-miR-4047-5p | SLC7A9 | L-cystine transport |
| L7 | cin-miR-4063-5p | SLC7A9 | L-cystine transport |
| L8 | cin-miR-4073-5p | SLC7A9 | L-cystine transport |
| T1 | cin-miR-124-2-5p | CYP2J2_1 | Tryptophan metabolism |
| T2 | cin-miR-2212b-5p | CYP2J2_1 | Tryptophan metabolism |
| T3 | cin-miR-4070-5p | CYP2J2_1 | Tryptophan metabolism |
| T4 | cin-miR-92f-5p | CYP2J2_1 | Tryptophan metabolism |
| T5 | cin-miR-196-5p | CYP2J2_1 | Tryptophan metabolism |
| T6 | cin-miR-184 | CYP4F2_1 | Tryptophan metabolism |
| T7 | cin-miR-35b-5p | CYP4F2_1 | Tryptophan metabolism |
| T8 | cin-miR-184 | CYP2J2_2 | Tryptophan metabolism |
| T9 | cin-miR-4086-3p | CYP2J2_2 | Tryptophan metabolism |
| T10 | cin-miR-4123-5p | CYP2J2_2 | Tryptophan metabolism |
| T11 | cin-miR-9880-3p | CYP2J2_2 | Tryptophan metabolism |
| T12 | cin-miR-5978a | CYP2J2_2 | Tryptophan metabolism |
| T13 | cin-miR-4036-5p | CYP2D6 | Tryptophan metabolism |
| T14 | cin-miR-4070-5p | CYP2D6 | Tryptophan metabolism |
| T15 | cin-miR-4073-5p | CYP2D6 | Tryptophan metabolism |
| T16 | cin-miR-4067-5p | CYP4F2_2 | Tryptophan metabolism |
| T17 | cin-miR-135-5p | CYP27B1 | Tryptophan metabolism |
| T18 | cin-miR-4044-5p | CYP27B1 | Tryptophan metabolism |
| T19 | cin-miR-4074-5p | CYP27B1 | Tryptophan metabolism |
| T20 | cin-miR-4163-5p | CYP27B1 | Tryptophan metabolism |
| T21 | cin-let-7c | CYP27B1 | Tryptophan metabolism |
| T22 | cin-miR-6111a-5p | CYP27B1 | Tryptophan metabolism |
| T23 | cin-miR-7707a-3p | CYP27B1 | Tryptophan metabolism |
| T24 | cin-miR-2212b-5p | CYP2U1 | Tryptophan metabolism |
| T25 | cin-miR-4000h-5p | CYP2U1 | Tryptophan metabolism |
| T26 | cin-miR-4079-5p | CYP7A1 | Tryptophan metabolism |
| T27 | cin-let-7c | CYP7A1 | Tryptophan metabolism |
| B1 | cin-miR-9311 | SRR | Biosynthesis of amino acids |
| B2 | cin-miR-9311 | GAPDH | Biosynthesis of amino acids |
| B3 | cin-miR-133-5p | GAPDH | Biosynthesis of amino acids |
| B4 | cin-miR-5978a | GAPDH | Biosynthesis of amino acids |
| B5 | cin-miR-7707a-3p | GAPDH | Biosynthesis of amino acids |
| B6 | cin-miR-135-5p | PGAM2_2 | Biosynthesis of amino acids |
| B7 | cin-miR-4000e-5p | PGAM2_2 | Biosynthesis of amino acids |
| B8 | cin-miR-4035-5p | PGAM2_2 | Biosynthesis of amino acids |
| B9 | cin-miR-6111a-5p | PGAM2_2 | Biosynthesis of amino acids |
| B10 | cin-miR-7433-5p | PGAM2_2 | Biosynthesis of amino acids |
| B11 | cin-miR-4001c-5p | PC | Biosynthesis of amino acids |
| B12 | cin-miR-4046-5p | PC | Biosynthesis of amino acids |
| Na1 | cin-miR-184 | SCN5A_3 | Na transport |
| Na2 | cin-miR-92d-5p | SCN5A_3 | Na transport |
| Na3 | cin-miR-4046-5p | SCN5A_4 | Na transport |
| Na4 | cin-miR-4123-5p | SCN5A_4 | Na transport |
| Na5 | cin-miR-4127-5p | SCN5A_4 | Na transport |
| Na6 | cin-miR-4046-5p | SCN9A_4 | Na transport |
| Na7 | cin-miR-rl1-6-3p | SCN9A_4 | Na transport |
| Na8 | cin-miR-4002-5p | SLC10A3 | Na transport |
| Na9 | cin-miR-133-5p | SLC10A3 | Na transport |
| K1 | cin-miR-183-5p | KCNA4 | K transport |
| K2 | cin-miR-92a-5p | KCNA4 | K transport |
| K3 | cin-miR-92d-5p | KCNA4 | K transport |
| K4 | cin-miR-92f-5p | KCNIP4_1 | K transport |
| K5 | cin-let-7c | KCNIP4_1 | K transport |
| K6 | cin-miR-4092-5p | KCNJ16 | K transport |
| K7 | cin-miR-11623-5p | KCNQ2 | K transport |
| K8 | cin-miR-4055-5p | KCNQ2 | K transport |
| K9 | cin-let-7b-5p | KCNK10 | K transport |
| K10 | cin-miR-4055-5p | KCNK10 | K transport |
| K11 | cin-miR-4001c-5p | KCNK18 | K transport |
| K12 | cin-miR-4045-5p | KCNK18 | K transport |
| K13 | cin-miR-4059-5p | KCNK18 | K transport |
| K14 | cin-miR-7707a-3p | KCNK5 | K transport |
| K15 | cin-miR-4000e-5p | KCNE5 | K transport |
| K16 | cin-miR-196-5p | KCNV1 | K transport |
| K17 | cin-miR-11623-5p | KCNQ2_1 | K transport |
| K18 | cin-miR-4055-5p | KCNQ2_1 | K transport |
| K19 | cin-miR-11623-5p | KCNQ2_2 | K transport |
| K20 | cin-miR-4055-5p | KCNQ2_2 | K transport |
| K21 | cin-miR-11623-5p | KCNQ2_3 | K transport |
| K22 | cin-miR-4055-5p | KCNQ2_3 | K transport |
| K23 | cin-miR-11623-5p | KCNQ2_4 | K transport |
| K24 | cin-miR-4055-5p | KCNQ2_4 | K transport |
| K25 | cin-miR-11623-5p | KCNQ2_5 | K transport |
| K26 | cin-miR-4055-5p | KCNQ2_5 | K transport |
| K27 | cin-miR-11623-5p | KCNQ2_7 | K transport |
| K28 | cin-miR-4055-5p | KCNQ2_7 | K transport |
| K29 | cin-miR-11623-5p | KCNQ2_8 | K transport |
| K30 | cin-miR-4055-5p | KCNQ2_8 | K transport |
| K31 | cin-miR-4067-5p | KCNIP1 | K transport |
| K32 | cin-miR-4092-5p | KCNIP1 | K transport |
| K33 | cin-miR-7707a-3p | KCNIP1 | K transport |
| K34 | cin-miR-4000h-5p | KCNQ3 | K transport |
| K35 | cin-miR-rl1-6-3p | KCNQ3 | K transport |
| K36 | cin-miR-4076-5p | KCNQ3 | K transport |
| K37 | cin-miR-4000b-5p | KCNK16 | K transport |
| K38 | cin-miR-7707a-3p | KCNK16 | K transport |
| K39 | cin-miR-4163-5p | HCN1 | K transport |
| K40 | cin-miR-4123-5p | KCNH3 | K transport |
| K41 | cin-miR-4163-5p | KCNS2 | K transport |
| K42 | cin-miR-872-3p | KCNS2 | K transport |
| K43 | cin-miR-92f-5p | KCNS2 | K transport |
| K44 | cin-miR-4073-5p | KCNS2 | K transport |
| K45 | cin-miR-4000b-5p | KCNS1 | K transport |
| Cl1 | cin-miR-4000e-5p | CLCNKA | Cl transport |
| Cl2 | cin-miR-7707a-3p | CLCNKA | Cl transport |
| Cl3 | cin-miR-4067-5p | CLCN6 | Cl transport |
| Cl4 | cin-miR-92d-5p | CLCN6 | Cl transport |
| Cl5 | cin-miR-4063-5p | SLC9B1P1 | Cl transport |
| Cl6 | cin-miR-1502b-5p | CLIC5 | Cl transport |
| Cl7 | cin-miR-4053-5p | CLIC5 | Cl transport |
| Cl8 | cin-miR-4055-5p | CLIC5 | Cl transport |
| Ca1 | cin-miR-4163-5p | CACNA1A | Ca transport |
| Ca2 | cin-miR-92d-5p | CACNA1E | Ca transport |
| Ca3 | cin-let-7b-5p | CACNG3 | Ca transport |
| Ca4 | cin-miR-4074-5p | CACNG3 | Ca transport |
| Ca5 | cin-miR-7433-5p | CACNG3 | Ca transport |
| Ca6 | cin-let-7b-5p | CACNA1H | Ca transport |
| Ca7 | cin-miR-4000e-5p | CACNA1I | Ca transport |
| Ca8 | cin-miR-4000h-5p | CACNA1I | Ca transport |
| Ca9 | cin-miR-5600-5p | TRPA1 | Ca transport |

**Table S3** The DNA methylation gene that regulated by miRNAs

| miRNA | Target | binding site | expression correlation |
| --- | --- | --- | --- |
| cin-miR-4059-5p | MBD2 | CDS | negative |
| cin-miR-92f-5p | MBD2 | CDS | positive |
| cin-miR-4046-5p | MBD2 | 3'UTR | positive |

**Table S4** The key gene during miRNA biogenesis and decay processes influenced by DNA methylation

| Gene ID | Gene name | Methy site | Location | Diff.methy |
| --- | --- | --- | --- | --- |
| KY.Chr5.678 | DGCR8 | Chr5:4487110 | gene body | up |
| KY.Chr5.678 | DGCR8 | Chr5:4488384 | gene body | up |
| KY.Chr5.678 | DGCR8 | Chr5:4481421 | gene body | up |

**Table S5** The GO terms enriched by dual-regulated genes

| Ontology | ID | Description | *p. adj* |
| --- | --- | --- | --- |
| MF | GO:0003676 | nucleic acid binding | 0.709 |
|  | GO:1901363 | heterocyclic compound binding | 0.709 |
| BP | GO:0015074 | DNA integration | 0.053 |
|  | GO:0006259 | DNA metabolic process | 0.618 |
|  | GO:0090304 | nucleic acid metabolic process | 0.796 |
